# Laparoscopic Delivery of a MnO_2_‐P‐ICG Patch for Photodynamic Therapy and NK Cell‐Driven Immunotherapy in Hepatocellular Carcinoma

**DOI:** 10.1002/advs.202524246

**Published:** 2026-04-03

**Authors:** Jie Lin, Haoqi Pan, Ke Wu, Junjie Nan, Jinyao Dai, Yushun Chang, Hao Shen, Qingxuan Ye, Haowen Lu, Yuxuan Shen, Boqiang Liu, Ming Wu, Jicheng Yu, Xiujun Cai, Dong Cen

**Affiliations:** ^1^ Department of General Surgery Sir Run‐Run Shaw Hospital Zhejiang University Hangzhou China; ^2^ National Engineering Research Center of Innovation and Application of Minimally Invasive Instruments Sir Run‐Run Shaw Hospital Zhejiang University Hangzhou China; ^3^ Department of Pancreatic Surgery Fudan University Shanghai Cancer Center Shanghai China; ^4^ State Key Laboratory for Diagnosis and Treatment of Infectious Diseases National Clinical Research Center for Infectious Diseases The First Affiliated Hospital Zhejiang University School of Medicine Hangzhou China; ^5^ Hepatobiliary Technology Key Laboratory of Fujian Province Mengchao Hepatobiliary Hospital of Fujian Medical University Fuzhou China; ^6^ State Key Laboratory of Advanced Drug Delivery and Release Systems Zhejiang Key Laboratory of New Drug Intelligent Innovation For Metabolic Diseases School of Pharmacy Zhejiang University Hangzhou China

**Keywords:** cancer immunotherapy, drug delivery, laparoscopic delivery, natural killer cells

## Abstract

Recurrence and metastasis are the primary causes of mortality in hepatocellular carcinoma (HCC), primarily driven by an immunosuppressive tumor microenvironment. This study developed a multifunctional flexible patch (MnO_2_‐P‐ICG NFs) based on electrospun SiO_2_ gel fibers whose surface is modified with a layer of “nest‐like” MnO_2_. The patch enables the co‐delivery of indocyanine green (ICG) and pachymaran directly to the tumor site via a laparoscopic system. Under near‐infrared light irradiation, ICG serves as a photosensitizer for photodynamic therapy (PDT), effectively ablating tumors and inducing immunogenic cell death (ICD), thereby activating antitumor immunity. Meanwhile, pachymaran, with its immunomodulatory effects, preferential activates natural killer (NK) cells to exert antitumor effects. Additionally, PDT further enhances this immune response by activating the cGAS‐STING pathway. In mouse models, implantation of the patch significantly inhibited primary liver tumor progression and ascites formation. In a malignant liver tumor model, MnO_2_‐P‐ICG NFs prevented local recurrence in 80% of treated mice. Notably, both the depletion of NK cells and the blockade of the cGAS‐STING pathway compromised the therapeutic efficacy of MnO_2_‐P‐ICG NFs. Clinically, tumors with higher NK cell infiltration were associated with improved patient outcomes. In conclusion, this versatile patch provides a promising therapeutic strategy for patients with advanced HCC.

## Graphical abstract

This study proposes a flexible MnO_2_‐P‐ICG nanofiber patch for laparoscopic treatment of liver tumors. The patch alleviates tumor hypoxia and induces photodynamic therapy (PDT) triggered immunogenic cell death, thereby activating the cGAS‐STING pathway and, in synergy with pachymaran, promoting NK cell–mediated innate immunity. This synergistic strategy effectively suppresses tumor growth, reduces ascites, and prevents tumor recurrence.

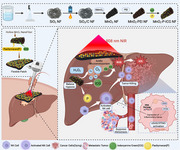

## 1 Introduction

Hepatocellular carcinoma (HCC) remains a formidable challenge globally, with rising incidence and mortality rates reported annually [1]. Projections suggest that by 2025, the global incidence of HCC will surpass one million cases [2]. The immune milieu in the liver is predominated by immunosuppressive cells and signals, cultivating a physiological state of tolerance necessary for liver function [3]. This immune microenvironment is maintained by a resident cell population [4]. In recent years, rapidly advancing immunotherapy has emerged as a promising cancer treatment owing to its favorable safety profile and durable therapeutic effects. Natural killer (NK) cells, a key component of the innate immune system, are particularly effective at triggering non‐antigen‐dependent immune responses against malignant tumors, thereby playing a critical role in cancer immunotherapy [5]. In the liver, NK cells are present at relatively high proportions, reflecting their crucial involvement in HCC progression [6, 7]. NK cells eliminate virus‐infected cells and malignant cells in the liver and inhibit liver fibrosis through the elimination of hepatic stellate cells. However, compared with non‐tumor tissues, the proportion of NK cells in tumor tissues of HCC patients is significantly reduced [8, 9, 10], and their functional impairment is associated with poor clinical outcomes [11]. These observations underscore the urgent need to restore NK cell–mediated antitumor immunity through therapeutic interventions. A comprehensive understanding of the hepatic microenvironment's intricate cellular and molecular immune networks will contribute to advancing innovative therapeutic approaches, including personalized and localized immunotherapies, for more effective management of primary and secondary liver cancer.

Studies have shown that traditional Chinese medicine, notably pachymaran (P), a key bioactive compound derived from poria cocos, can stimulate the innate immune system to combat malignant tumors. Pachymaran enhances the activity of innate immune cells, boosting their phagocytic or cytotoxic capabilities and modulating broader immune responses. When combined with chemotherapeutic agents, pachymaran can further augment antitumor effects [12, 13]. However, pharmacokinetic limitations necessitate the development of a new drug delivery system to optimize the clinical application of pachymaran.

Biomaterials as a drug delivery system (DDS) have become a significant focus in tumor therapy. In particular, advances in nanomaterials and related technologies have led to their widespread application in biomedicine, especially in cancer treatment [14]. Nanomaterial‐based tumor therapy strategies have introduced innovative approaches with clinical translational potential, such as photodynamic therapy (PDT) [15, 16]. PDT involves laser irradiation at a specific wavelength to activate a photosensitizer, which transfers energy to molecular oxygen to generate reactive oxygen species (ROS) that damage tumor cells [17, 18]. PDT not only eradicates tumors but also induces immunogenic cell death (ICD) in cancer cells, leading to the release of tumor‐associated antigens and proinflammatory cytokines that stimulate antitumor immune responses [19]. Recent studies have highlighted the role of metal ions in the tumor microenvironment, where they catalyze reactions with hydrogen peroxide to generate oxygen [20]. This oxygen generation alleviates tumor hypoxia and improves the efficacy of hypoxia‐limited PDT [21]. Additionally, metal ions play a crucial role in the cyclic GMP‐AMP synthase/stimulator of interferon genes (cGAS‐STING) signaling pathway, promoting innate immune responses and resisting infections from pathogenic microorganisms, including viruses [22, 23]. Activation of the cGAS‐STING signaling pathway has emerged as a promising strategy to enhance tumor sensitivity to immunotherapy [24, 25, 26]. Although nanodrug delivery systems can serve as integrated platforms to incorporate multiple functional components and improve therapeutic efficacy, their accumulation efficiency at tumor sites remains suboptimal. At the same time, off‐target distribution continues to cause unavoidable toxic side effects.

Based on the above rationale, we designed a versatile patch as a local drug delivery system (LDDS) to deliver pachymaran, MnO_2_, and indocyanine green (ICG), enabling targeted PDT and immunotherapy at the tumor site via laparoscopic delivery. The system utilizes hollow MnO_2_ nanofibers synthesized through a multistep process involving electrospinning of silica gel fibers, nitrogen sintering, and carbon reduction of potassium permanganate. Further modification was achieved by electrostatic adsorption of pachymaran and the photosensitizer (ICG onto the MnO_2_ NFs, yielding a composite drug delivery system termed MnO_2_‐P‐ICG nanofibers (MnO_2_‐P‐ICG NFs). This system is designed to facilitate localized tumor delivery, thereby activating NK cells to exert antitumor effects through PDT and activation of the cGAS‐STING pathway.

## 2 Results

### 2.1 Pachymaran Regulates the Immune Microenvironment and Modulates Tumor Cell Migration

Previous studies have shown that the traditional Chinese medicine pachymaran exhibited antitumor activity, modulates the immune environment, enhances nonspecific immune function, and promotes NK cell activation [27]. These properties highlight its considerable potential in cancer treatment, particularly as an adjuvant therapy. However, the mechanisms underlying its antitumor and immunomodulatory effects remain unclear. In this study, we investigated the mechanisms by which pachymaran modulates tumor immunity using both in vivo and in vitro models.

**FIGURE 1:**
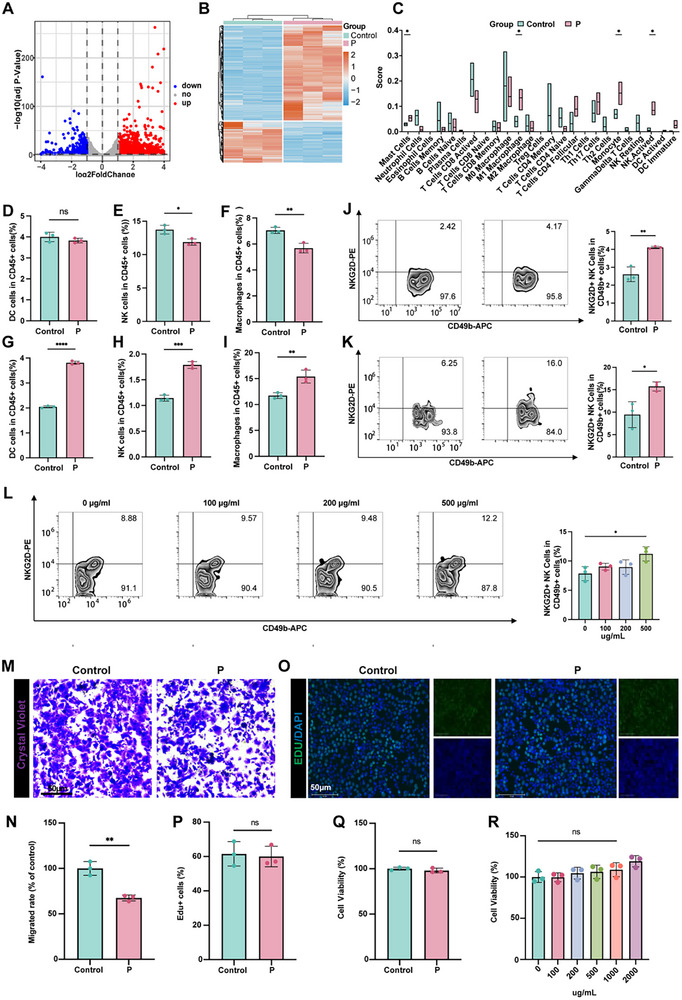
The effect of pachymaran on the immune environment, growth and migration of mouse liver cancer cells. (A) Volcano plot of pachymaran versus Control (|Fold Change| > 2, *p* < 0.05). (B) Heatmap of differentially expressed genes (*n *= 3/group). (C) Box plots of immune cell scores (*n *= 3/group). (D–F) Effects of pachymaran on DCs (*n *= 3/group) (D), NK cells (*n *= 3/group) (E), and macrophages (*n* = 3/group) (F) in the spleen of mice. (G–I) Effects of pachymaran on DCs (*n *= 3/group) (G), NK cells (*n *= 3/group) (H), and macrophages (*n *= 3/group) (I) in the peritoneal lymph nodes of mice. (J) Effect of pachymaran on the activation ratio of NK cells in the spleen of mice (*n *= 3/group). (K) Effect of pachymaran on the activation ratio of NK cells in the peritoneal lymph nodes of mice (*n *= 3/group). (L) Effect of pachymaran on the activation ratio of primary NK cells in vitro (*n *= 3/group). (M, N) Transwell assay results demonstrating the effect of pachymaran on the migration capability of tumor cells (scale bar = 50 µm, *n *= 3/group); (O, P) EdU staining assays showing the effect of pachymaran on the proliferation capability of tumor cells (scale bar = 100 µm, *n *= 3/group). (Q) Effect of pachymaran on the proliferation capability of tumor cells (*n *= 3/group). (R) Effect of different concentrations of pachymaran on the proliferation of tumor cells (*n *= 3/group). Data were presented as mean ± SD. Unpaired t‐test and ordinary one‐way ANOVA were performed, *p < 0.05, **p < 0.01, ***p < 0.001, ****p < 0.0001, ns = not significant.

First, we assessed the effects of pachymaran on antitumor efficacy and the intratumoral immune milieu by administering it via intraperitoneal injection. After a two‐week treatment period, pachymaran treatment significantly inhibited the ascites metastasis of H22 tumor cells (Figure S1A). Subsequently, the mice were euthanized, and tumor specimens were harvested for RNA sequencing. The data are provided in Data S1. As shown in Figure 1A, pachymaran treatment induced a total of 2459 differentially expressed genes in tumor tissues, including 1698 genes upregulated and 761 genes downregulated. The heatmap of these differentially expressed genes is depicted in Figure 1B. We further analyzed immune cell infiltration in mouse tumor tissues using the CIBERSORT online tool (https://cibersortx.stanford.edu/). The gene signature matrix for murine immune cell profiling was obtained from Z. et al. [28]. As shown in Figure 1C, pachymaran‐treated tumors exhibited increased proportions and infiltration scores of innate immune cells, with notable elevations in activated NK cells, M1‐type macrophages, and monocytes.

To further verify the immunomodulatory effects of pachymaran in vivo, mice received intraperitoneal injections of either pachymaran or PBS every two days for one week. After euthanasia, spleens and abdominal lymph nodes were harvested, and the isolated cells were stained for flow cytometry analysis. The flow cytometry gating strategy is shown in Figure S1B. As shown in Figure 1D–F and Figure S1C–G, the proportions of dendritic cells (DCs), NK cells, macrophages, and T cells in the spleen were not significantly altered. However, Figure 1G–I and Figure S1H–J show that pachymaran increased the proportion of DCs, NK cells, and macrophages in the abdominal lymph nodes. By contrast, the proportion of T cells in the abdominal lymph nodes did not increase (Figure S1K,L). Additionally, analysis of NK cell activation revealed a significant increase in the proportion of NKG2D^+^ NK cells. Together, these results indicate that pachymaran modulates the distribution and activation of innate immune cells, with a pronounced effect on NK cell activation.

To assess the ability of pachymaran to activate NK cells, we isolated primary immune cells from the spleens of untreated mice and subsequently incubated them with different concentrations of pachymaran in vitro. After 8 h of incubation, the cells were collected for flow cytometry analysis to evaluate NK cell activation. The results showed that lower concentrations of pachymaran had no significant effect on NK cell activation. In contrast, treatment with pachymaran at 500 µg/mL significantly increased the proportion of activated NK cells (Figure 1L). These results indicate that pachymaran at an appropriate concentration enhances NK cell activation both in vivo and in vitro.

Subsequently, we examined the effects of pachymaran on tumor cell migration and proliferation. Transwell assay results demonstrated that pachymaran treatment significantly reduced the migratory capacity of tumor cells (Figure 1M,N). EdU staining analysis showed that pachymaran treatment alone did not significantly affect tumor cell proliferation (Figure 1O,P). Consistently, CCK‐8 assay results indicated that pachymaran treatment alone did not affect tumor cell proliferation (Figure 1Q,R). Collectively, these findings suggest that pachymaran does not substantially influence tumor cell proliferation but effectively suppresses tumor cell migration.

### 2.2 Construction and Characterization of MnO_2_‐P‐ICG NFs

**FIGURE 2:**
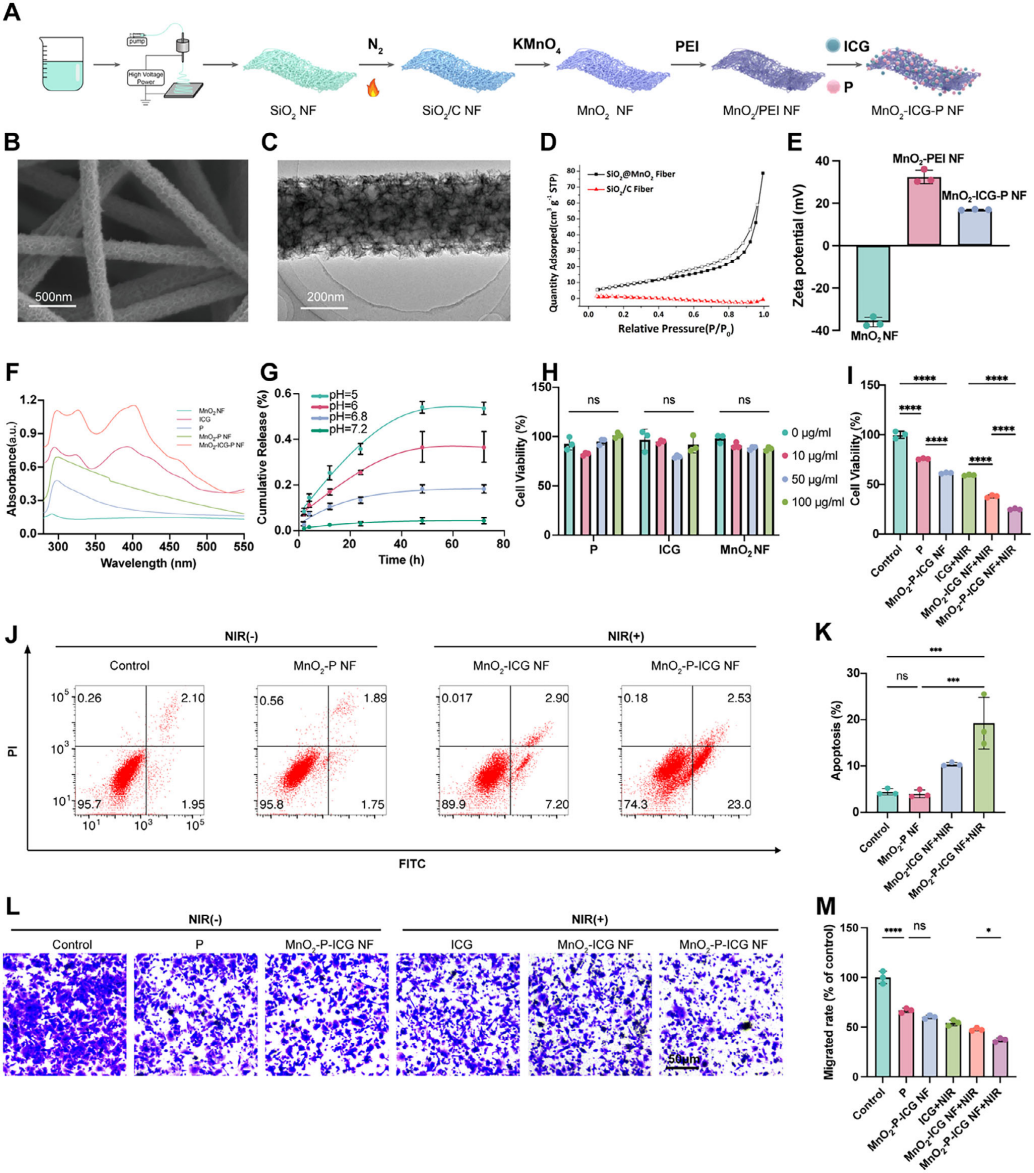
Synthesis and characterization of MnO_2_‐P‐ICG NFs and their effects on the growth, migration, and apoptosis of mouse liver cancer cells. (A) Synthesis schematic of the composite drug delivery system MnO_2_‐P‐ICG NFs loaded with pachymaran. (B, C) Microstructure characterization of MnO_2_ NFs: SEM image of hollow MnO_2_ NFs (scale bar: 500 nm) (B) and TEM image (scale bar: 20 nm) (C). (D) Surface area testing analysis. (E) Zeta potential analysis showing the potential changes during the preparation process of MnO_2_‐P‐ICG NFs (*n *= 3/group). (F) UV–vis absorption spectra showing characteristic peaks associated with drug loading in MnO_2_‐P‐ICG NFs. (G) pH‐dependent release of pachymaran from MnO_2_‐P‐ICG NFs (*n *= 3/group). (H) Effect of MnO_2_ NF, pachymaran, and ICG within the composite drug delivery system on the growth and proliferation of normal mouse liver cells (*n *= 3/group). (I) Effect of the composite drug delivery system under 808 nm NIR on the growth and proliferation of mouse liver cancer cells in a co‐culture system (*n* = 3/group). (J, K) Effect of the composite drug delivery system on apoptosis of mouse liver cancer cells (*n *= 3/group). (L, M) Effect of the composite drug delivery system on migration of liver mouse cancer cells (scale bar = 50 µm, *n *= 3/group). Data were presented as mean ± SD. Ordinary one‐way ANOVA was performed, *p < 0.05, **p < 0.01, ***p < 0.001, ****p < 0.0001, ns = not significant.

To construct the patch scaffold, silicon dioxide (SiO_2_) gel fibers were synthesized via the sol‐gel method using tetraethyl orthosilicate as the precursor, deionized water as the solvent, phosphoric acid as the catalyst, and polyvinyl alcohol as the spinning agent. We systematically adjusted hydrolysis reaction parameters, including the deionized water ratio, hydrolysis duration, and temperature, to generate precursor solutions with varying viscosity, surface tension, and conductivity. By fine‐tuning the electrospinning parameters, including applied voltage and flow rate, we successfully produced a series of SiO_2_ gel fibers with diameters ranging from 100 to 300 nm. These fibers were subsequently subjected to nitrogen sintering followed by potassium permanganate–mediated carbon reduction, yielding hollow manganese dioxide (MnO_2_) nanofibers, as depicted in Figure 2A.

Scanning electron microscopy (SEM) and transmission electron microscopy (TEM) analyses revealed that the MnO_2_ nanofibers exhibited a uniform hollow structure with diameters of approximately 200 nm (Figure 2B,C). Surface area analysis indicated that this porous architecture exhibited a higher specific surface area compared with silicon dioxide/carbon (SiO_2_/C) nanofibers without MnO_2_ decoration, rendering it more suitable for high‐capacity drug loading (Figure 2D). Furthermore, X‐ray diffraction (XRD) analysis confirmed that the phase composition of the hollow MnO_2_ nanofibers was consistent with that of standard MnO_2_ (Figure S2A).

To achieve the dual functions of immunotherapy and PDT, pachymaran and ICG were successfully loaded onto hollow MnO_2_ nanofibers via electrostatic adsorption. Zeta potential analysis revealed that the hollow MnO_2_ nanofibers exhibited a negative charge. Upon incubation with a polyethyleneimine (PEI) solution, the nanofibers acquired a positive surface charge, as shown in Figure 2E. Subsequent loading of pachymaran and ICG onto the PEI‐modified MnO_2_ nanofibers led to a noticeable reduction in positive surface charge, indicating successful co‐loading of pachymaran and ICG onto the nanofibers (Figure 2E). UV–vis spectroscopy confirmed the effective loading of pachymaran by the presence of its characteristic absorption peaks in the MnO_2_‐P NFs spectrum. Moreover, the presence of distinct ICG absorption peaks in the MnO_2_‐P‐ICG NFs spectrum, compared with the MnO_2_‐P NFs, further confirmed the successful loading of ICG (Figure 2F). Optimization of the mixing ratios of pachymaran, ICG, and MnO_2_ nanofibers revealed that the optimal loading efficiency was achieved at a P/MnO_2_ NF ratio of 0.8 and an ICG/MnO_2_ NF ratio of 8, as illustrated in Figure S2B,C.

UV–Vis spectroscopy was employed to determine pachymaran concentrations to establish a standard curve for quantitative assessment (Figure S2D). The release of pachymaran from MnO_2_‐P‐ICG NFs under different pH conditions was investigated. Our findings indicated a marked increase in the release of pachymaran from MnO_2_‐P‐ICG NFs as pH decreased, indicating a pH‐sensitive release profile (Figure 2G). This result suggests that pachymaran can be effectively released in the acidic tumor microenvironment. The oxygen production capacity of MnO_2_‐P‐ICG NFs was evaluated using a dissolved oxygen meter. It was observed that MnO_2_‐P‐ICG NFs produced minimal oxygen under physiological conditions. However, upon exposure to elevated H_2_O_2_ levels, the oxygen yield within the MnO_2_‐P‐ICG NFs system increased substantially, indicating its potential to react with the high levels of H_2_O_2_ in the tumor microenvironment to generate oxygen (Figure S2E). To directly evaluate the effect of MnO_2_ on tumor hypoxia, HIF‐1α protein expression was assessed at both the cellular level and in tumor tissues. As shown in the results, MnO_2_‐based treatment significantly reduced HIF‐1α expression in vitro and in tumor tissues, indicating effective alleviation of the hypoxic tumor microenvironment (Figure S3A,B). This oxygen generation could help alleviate tumor hypoxia and enhance the efficacy of subsequent PDT.

The PDT efficacy under 808 nm NIR irradiation was evaluated using 1,3‐diphenylisobenzofuran (DPBF) degradation assay to detect ROS generation. In the control group, DPBF remained stable (Figure S2F). Similarly, in the MnO_2_‐P‐ICG NFs system in the absence of H_2_O_2_, DPBF degradation was negligible (Figure S2G). In contrast, the introduction of H_2_O_2_ into the MnO_2_‐P‐ICG NFs system led to near‐complete DPBF degradation within 270 s (Figure S2H), indicating effective ROS generation and highlighting the system's PDT potential under NIR irradiation in the presence of H_2_O_2_.

### 2.3 In Vitro Antitumor Therapy of MnO_2_‐P‐ICG NFs

Before evaluating the biosafety of the final product, MnO_2_‐P‐ICG NFs, we first analyzed the impact of each component on cell viability using the CCK‐8 assay. As shown in Figure 2H, a range of concentrations of pachymaran and ICG exerted negligible effects on the viability of mouse hepatocytes. Similarly, MnO_2_ NFs at concentrations below 100 µg/mL showed no significant cytotoxicity. However, when the concentration exceeded 100 µg/mL, the cytotoxicity of MnO_2_ NF increased in a concentration‐dependent manner. These results indicate that although MnO_2_ NFs exhibit measurable cytotoxicity at higher concentrations, they demonstrate good biocompatibility within an appropriate dosage range. Based on these findings, we selected 100 µg/mL as the working concentration of MnO_2_ NFs for subsequent experiments.

The in vitro efficacy of PDT against cancer cells was evaluated using the CCK‐8 assay. In the absence of ICG, 808 nm NIR irradiation alone did not affect the growth of mouse liver cancer cells, and no PDT effect was observed. Subsequently, mouse hepatocytes and immune cells were exposed to NIR irradiation at various power densities and exposure times, and the optimal treatment parameters were determined to be 1 W/cm^2^ for 120 s, respectively (Figure S2I–L).

To evaluate the efficacy of the MnO_2_‐P‐ICG NFs drug delivery patch on mouse hepatocellular carcinoma cells, we performed a series of in vitro experiments, including CCK‐8 assays, apoptosis assays, and Transwell assays. The cells were treated with culture media containing the respective factors at a concentration of 100 µg/mL for 6 h. For the NIR groups, cells were irradiated with 808 nm NIR light at a power density of 1 W/cm^2^ for 120 s after incubation. To better investigate the effects of MnO_2_‐P‐ICG NFs on immune cells and tumor cells, we established a co‐culture system of NK cells and adherent hepatocellular carcinoma cells to simulate the immune–tumor cell interactions and assess the functional impact of pachymaran treatment. This model provides a more physiologically relevant context in which the immunological effects of pachymaran can be evaluated. The results yielded two key observations (Figure 2I): First, pachymaran treatment alone significantly reduced the viability of tumor cells in the NK–tumor co‐culture system, which may be attributed to its immune‐activating properties. Second, the combination of pachymaran with MnO_2_‐ICG NF produced a more pronounced inhibitory effect on tumor cells compared with MnO_2_‐ICG NF alone. These findings suggest a synergistic effect, in which pachymaran‐activated NK cells enhance the antitumor efficacy of MnO_2_‐ICG NFs through immune‐mediated mechanisms in addition to photodynamic effects. Further analysis of the apoptosis rate in mouse hepatocellular carcinoma cells was conducted using an Annexin V‐FITC/PI apoptosis detection kit, followed by flow cytometry. As shown in Figure 2J,K, the results demonstrated that MnO_2_‐P‐ICG NFs significantly promoted the apoptosis of mouse hepatocellular carcinoma cells under 808 nm NIR irradiation, with a markedly higher apoptosis rate compared to other groups. This finding is consistent with the CCK‐8 assay results. Additionally, the impact on tumor cell migration was evaluated via Transwell migration assays. The results showed that the MnO_2_‐P‐ICG NF+NIR group significantly inhibited tumor cell migration, outperforming other groups in PDT‐mediated suppression (Figure 2L,M). These results suggest that the as‐prepared multifunctional patch can effectively inhibit tumor cell proliferation and migration and promote apoptosis, and these effects will be further validated in vivo.

### 2.4 The Effect of MnO_2_‐P‐ICG NF on Activating NK Cells In Vitro

NK cells are vital components of the innate immune system, typically identified in mice by the expression of surface markers such as NK1.1, NKp46, and CD49b, together with the absence of the T cell marker CD3 to exclude contaminating T cell subsets. The NKG2 receptor family is critically involved in NK cell function and includes NKG2A, NKG2B, NKG2C, NKG2D, NKG2E, and NKG2F. Among these, activating receptors play a vital role in mediating NK cell cytotoxicity against abnormal cells, with NKG2D being a key receptor for NK cell activation. NKG2D is expressed on NK cells as a homodimer and is widely utilized as an activation marker for NK cells. Previous sequencing data and flow cytometry analyses of primary immune cells indicate that pachymaran can promote the infiltration of innate immune cells, notably enhancing NK cell activation.

**FIGURE 3:**
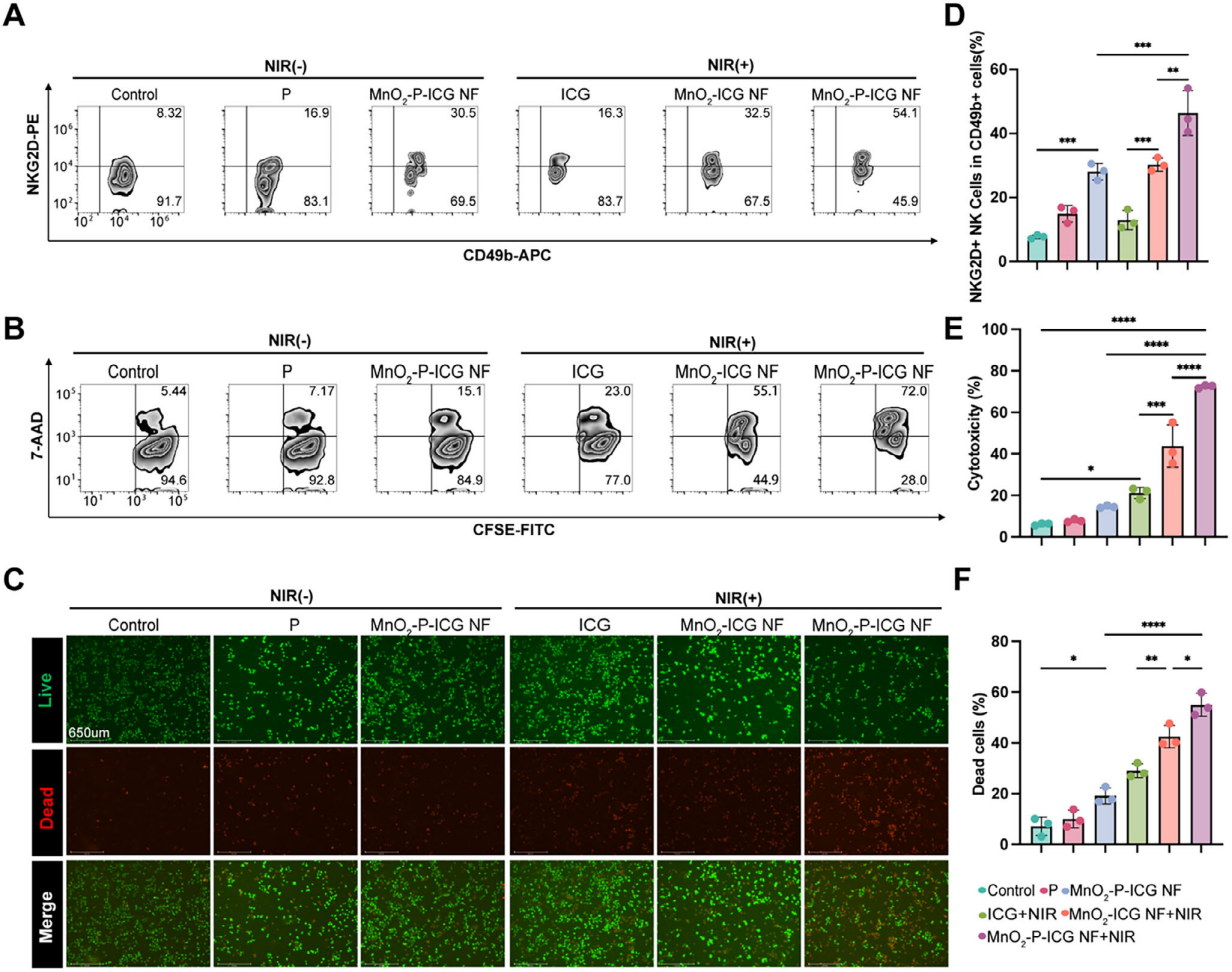
Enhancement of NK cell activation and cytotoxicity by composite drug delivery system MnO_2_‐P‐ICG NFs in vitro. (A, D) Flow cytometry analysis of the activation effect of the composite drug delivery system MnO_2_‐P‐ICG NFs on NK cells (*n *= 3/group). (B, E) Flow cytometry analysis of the effect of the composite drug delivery system on NK cell cytotoxicity (*n *= 3/group). (C, F) Live/Dead staining showing the proportion of target cell death mediated by NK cells following treatment with the composite drug delivery system (scale bar = 650 µm, *n *= 3/group). Data were presented as mean ± SD. Ordinary one‐way ANOVA was performed, *p < 0.05, **p < 0.01, ***p < 0.001, ****p < 0.0001.

To further validate whether MnO_2_‐P‐ICG NFs could promote the activation of innate immune cells, particularly NK cells, in vitro, we used flow cytometry to examine their effects. NK cells were identified as CD45^+^CD3‐CD49b^+^ cells, and activated NK cells were labeled using an anti‐NKG2D antibody. As shown in Figure 3A,D, our results revealed that, even in the absence of PDT, MnO_2_‐P‐ICG NFs significantly increased the activation rate of NK cells. Furthermore, under 808 nm NIR‐induced PDT, this patch further enhanced NK cell activation compared to conditions without PDT. Notably, compared to its counterpart lacking pachymaran, MnO_2_‐P‐ICG NFs exhibited a more pronounced activation effect on NK cells.

When NK cells are co‐cultured with mouse liver cancer Hepa1‐6 cells, they exert measurable inhibitory and cytotoxic effects on Hepa1‐6 cells. Since the co‐culture system includes both effector and target cells, CFSE dye was used to label Hepa1‐6 cells, and CD49b was used as a specific surface marker for NK cells. We employed 7‐aminoactinomycin D (7‐AAD) as a single viability stain to identify dead cells, as a membrane‐impermeable nucleic acid dye, 7‐AAD binds to DNA in cells with compromised membrane integrity, allowing specific identification of killed target cells. Utilizing flow cytometry and immunofluorescence analyses, we confirmed the ability of MnO_2_‐P‐ICG NFs to enhance NK cell cytotoxicity. Following the aforementioned grouping and treatment procedures, Hepa1‐6 cells were co‐cultured with primary NK cells for 8 h, after which both supernatant and adherent cells were collected, washed, centrifuged, resuspended, and stained with flow cytometry antibodies to measure the proportion of killed Hepa1‐6 cells. These results demonstrated that MnO_2_‐P‐ICG NFs significantly potentiated NK cell‐mediated cytotoxicity toward target cells. Notably, in the absence of pachymaran, PDT‐induced NK cell cytotoxicity was markedly reduced. Moreover, even in the absence of PDT, our composite drug delivery system alone still enhanced NK cell‐mediated killing of target cells (Figure 3B,E).

Subsequently, we further validated the promoting effect of the composite drug delivery system on NK cell‐mediated killing toward target cells using a dual‐color fluorescence‐based cell viability assay. The results demonstrated that the composite drug delivery system significantly enhanced NK cell‐mediated killing of target cells. Moreover, this system further augmented NK cell‐mediated killing toward target cells under PDT, consistent with the flow cytometry results (Figure 3C,F). Taken together, pachymaran and ICG‐induced PDT not only stimulated NK cell activation but also enhanced their cytotoxic effects on target cells, indicating a synergistic interaction between pachymaran and PDT.

### 2.5 Extensibility, Distribution, Biocompatibility, and Uptake of MnO_2_‐P‐ICG NFs

**FIGURE 4:**
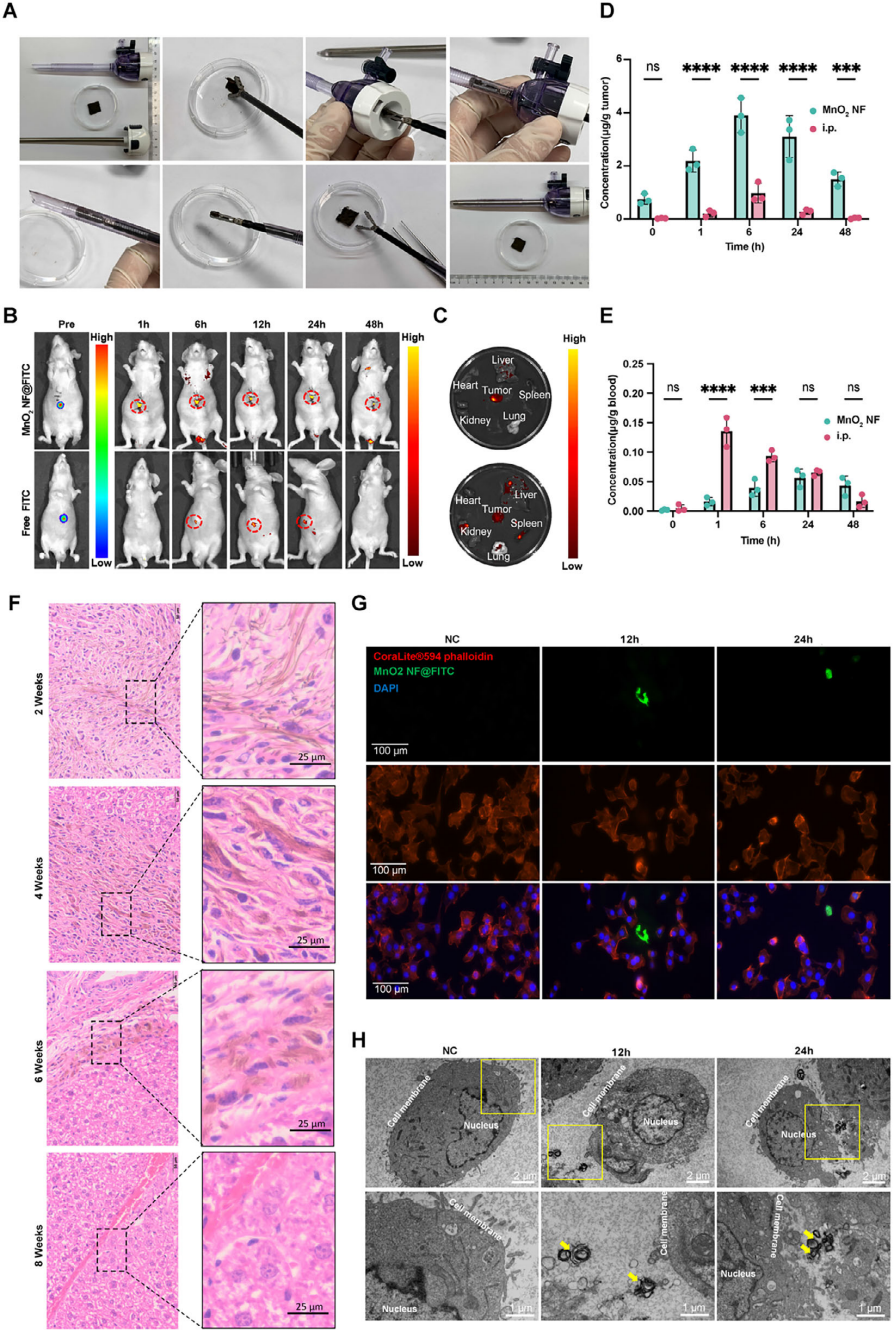
Adhesive properties and biocompatibility of the MnO_2_ NF carrier for the composite drug delivery system in vivo. (A) Demonstrating the extensibility of MnO_2_ NF under endoscopic simulation conditions. (B) Time‐dependent in vivo fluorescence images of FITC‐labeled MnO_2_ NF in orthotopic Hepa1‐6 tumors in mice. (C) Ex vivo fluorescence images of tumor and major organs 48 h after MnO_2_ NF treatment. (D) Plasma drug concentration at different time points after treatment (*n *= 3/group). (E) Intratumoral drug concentration at different time points after treatment (*n *= 3/group). (F) Metabolism of MnO_2_ NF in vivo revealed by H&E pathological staining of tissue sections (scale bar = 50 µm). (G) Fluorescence images of MnO_2_ NF cellular uptake by Hepa1‐6 cells at different time points (scale bar = 100 µm). (H) Cryo‐TEM images of MnO_2_ NF cellular uptake by Hepa1‐6 cells at different time points (scale bar 1 = 2 µm, scale bar 2 = 1 µm). Data were presented as mean ± SD. Two‐way ANOVA was performed, ***p < 0.001, ****p < 0.0001, ns = not significant.

The extensibility and adhesion of curative patches are essential for ensuring the proper placement and sustained release at the target site. Meanwhile, minimally invasive surgery represents a significant trend in modern surgical practice. To address these requirements, we conducted simulations under laparoscopic conditions to assess the extensibility of the as‐prepared MnO_2_‐P‐ICG NFs, thereby laying the groundwork for their application in drug delivery. As depicted in Figure 4A, the drug‐loaded patches were successfully grasped by forceps, curled, passed through a 10 mm laparoscopic trocar, and then smoothly returned to their original shape upon manipulation with forceps. Additionally, MnO_2_ NFs labeled with FITC fluorophores were applied to the surfaces of hepatic tumors in mice to further investigate their distribution in vivo. Compared with intraperitoneal injection, locally applied MnO_2_ NF@FITC showed significant accumulation at the tumor site and persisted for a longer duration (Figure 4B and Figure S4). Even 48 h after organ and tumor harvest, the fluorescence signal remained prominent, primarily localizing in the tumor and liver rather than being systemically distributed (Figure 4C). In addition, following treatment with MnO_2_ NF@P, drug concentrations in the tumor and plasma were measured at different time points, revealing that MnO_2_ NF@P increased intratumoral drug accumulation while maintaining lower plasma drug levels (Figure 4D,E).

Biocompatibility is defined as a material's capacity to interact with biological systems without provoking adverse reactions and to maintain a neutral physiological impact on the organism upon contact. It serves as a pivotal criterion for evaluating the suitability of nanomaterials for clinical translation. To evaluate biocompatibility, we conducted histopathological analyses and monitored longitudinal changes in mouse body weight. As shown in Figure 4F, H&E staining revealed that MnO_2_ NFs did not induce significant tissue necrosis or inflammatory responses in vivo. At two weeks post‐implantation, MnO_2_ NFs retained a distinct fibrous structure; by four and six weeks, progressive cellular infiltration and metabolic degradation were observed, and the material was almost completely metabolized by eight weeks. Furthermore, as illustrated in Figure S5A, no significant differences in body weight changes were observed between the MnO_2_ NFs‐implanted and control groups. Collectively, these results confirm the excellent in vivo biocompatibility of MnO_2_ NFs, supporting their potential as viable drug delivery carriers. In addition, fluorescence imaging and TEM analyses indicated that the nanofibers were not readily internalized by cells. As shown in Figure 4G, MnO_2_@FITC exhibited minimal cellular internalization following co‐incubation, and TEM observations further supported this finding (Figure 4H). Based on these findings, the drug delivery system is proposed to primarily release therapeutic agents into the tumor microenvironment, thereby exerting therapeutic effects without extensive cellular phagocytosis.

To assess the in vivo biosafety of the composite drug delivery system, we collected heart, liver, spleen, lung, kidney, and serum samples from mice in each group following treatment. No significant tissue necrosis or inflammatory responses were observed in the treated groups based on H&E‐stained histopathological sections of major organs (Figure S5B). Additionally, ALT and AST showed no significant differences between the control and system‐treated groups, indicating preserved liver function (Figure S5C). Taken together with the stable body weight profiles observed during the treatment period, these findings indicate that the composite drug delivery system exhibits good biosafety.

### 2.6 In Vivo Antitumor Effect of MnO_2_‐P‐ICG NFs

**FIGURE 5:**
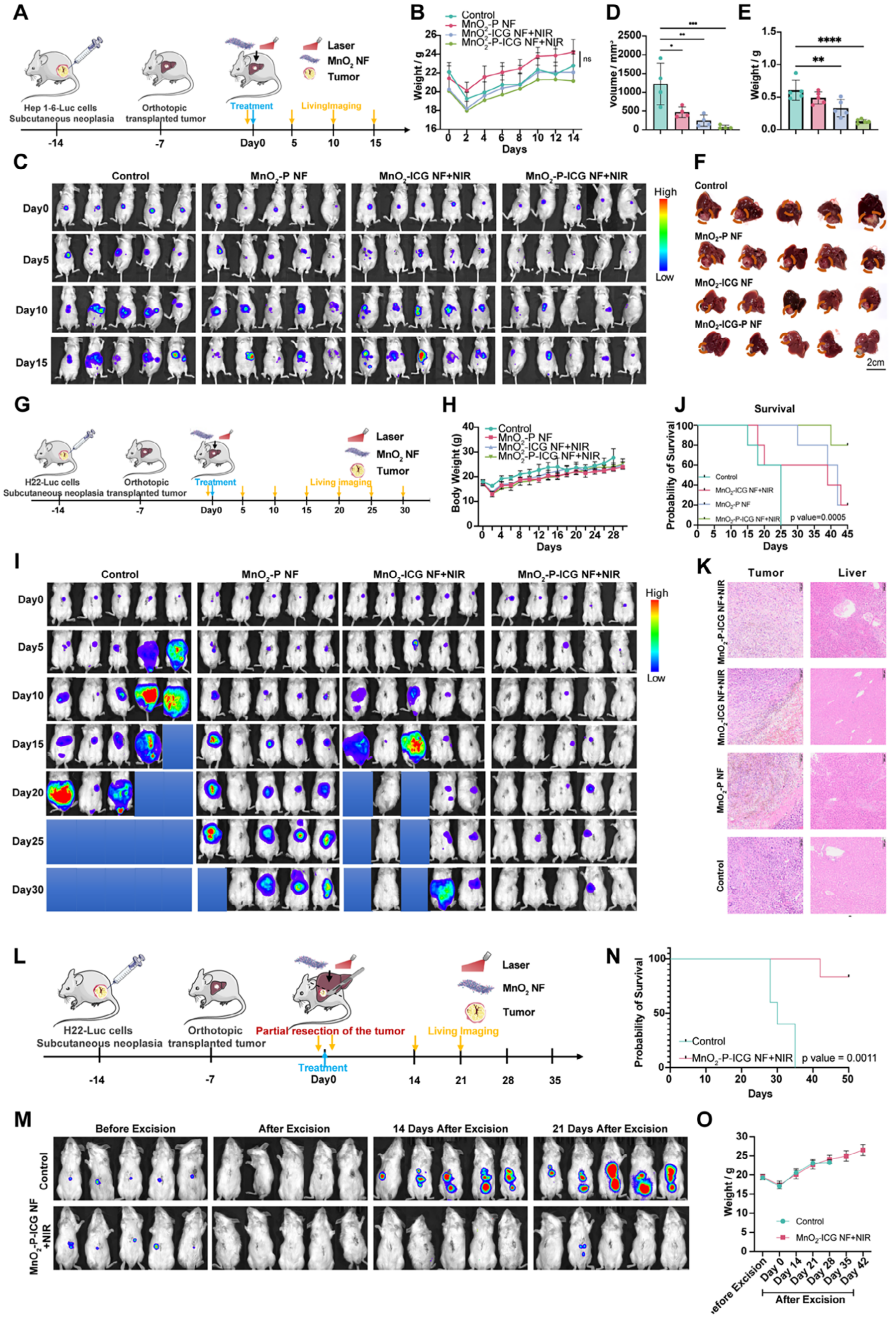
Antitumor effects of the composite drug delivery system MnO_2_‐P‐ICG NFs in vivo. (A) Schematic diagram of the in vivo antitumor therapy process. (B) Changes in body weight of mice during the treatment period (*n *= 5/group). (C) In vivo live imaging showing the growth of orthotopic tumors in mice during treatment. (D) Tumor volume of mice post‐treatment (*n *= 5/group). (E) Tumor weight of mice post‐treatment (*n* = 5/group). (F) Gross images of orthotopic tumors in mice from each group post‐treatment. (G) Schematic diagram of the in vivo anti‐metastatic therapy process. (H) Changes in body weight of mice during the treatment period (*n *= 5/group). (I) In vivo fluorescence images showing the growth of orthotopic tumors and ascites formation in mice during treatment. (J) Survival curves of mice in each group at day 45 post‐treatment (*n *= 5/group). (K) Representative images of H&E staining of tumors and adjacent tissues in mice from each group (scale bar 1 = 200 µm, scale bar 2 = 100 µm). (L) Schematic diagram of the in vivo anti‐recurrence therapy process. (M) In vivo fluorescence images showing the recurrence of orthotopic tumors in mice during treatment. (N) Survival status of mice in each group at day 50 post‐treatment (*n *= 5/group). (O) Changes in body weight of mice during the treatment period (*n *= 5/group). Data were presented as mean ± SD. Ordinary one‐way ANOVA and log‐rank test were performed, *p < 0.05, **p < 0.01, ***p < 0.001, ****p < 0.0001.

To validate the in vivo antitumor efficacy of MnO_2_‐P‐ICG NFs, we followed the procedure outlined in Figure 5A. First, we established subcutaneous Hepa1‐6 tumor models. Once the tumors reached a predetermined size, they were harvested and used to establish Hepa1‐6 orthotopic tumor models. Successful tumor establishment was confirmed by live imaging, after which the mice were randomly assigned to four groups. Drug‐loaded patches incorporating responsive functional components were then applied topically to the tumors, and the last two groups additionally received 808 nm NIR irradiation as specified. Body weights were recorded every two days following treatment, while tumor progression was monitored via live imaging every five days. At the end of the 15‐day treatment period, the mice were euthanized, and tumor tissues were harvested for volume measurement and weighing.

As depicted in Figure 5B, all groups of mice experienced a temporary weight loss following surgery, but body weight changes during the treatment period were comparable between the treatment and the control groups, indicating that MnO_2_‐P‐ICG NFs as a composite drug delivery system did not induce systemic toxicity. Live imaging results showed that the composite drug delivery system containing both pachymaran and ICG, when combined with NIR laser irradiation, exhibited superior therapeutic efficacy at five days post‐treatment compared to its counterparts containing either pachymaran or ICG under the same NIR laser conditions (Figure 5C). The tumor volume and tumor weight measured at the end of treatment led to similar conclusions, as shown in Figure 5D–F and Figure S6.

To demonstrate the inhibitory effect of the patch on liver tumor metastasis, we used the highly aggressive H22 cell line to establish an orthotopic liver cancer model characterized by hepatic ascites in BALB/c mice. The experimental procedure is outlined in Figure 5G. We systematically documented tumor growth, the incidence of distant metastasis accompanied by abdominal effusion, and survival duration for each mouse group. Analysis of body weight changes over the 30 days showed that the composite drug delivery system did not induce significant weight loss, indicating minimal systemic toxicity during long‐term treatment (Figure 5H). Over a 30‐day observation period, MnO_2_‐P NF treatment significantly inhibited distant tumor metastasis and the formation of extensive abdominal effusion compared with the control group; however, its suppressive effect on primary tumor growth was relatively modest. In contrast, MnO_2_‐ICG NF treatment efficiently suppressed tumor growth through PDT but exhibited weak inhibition of distant metastasis and effusion formation. In sharp contrast, MnO_2_‐P‐ICG NF, in combination with PDT, markedly inhibited both primary tumor growth and distant metastasis, along with extensive effusion formation (Figure 5I). Additionally, macroscopic examination of liver specimens revealed that pachymaran‐loaded treatment groups exhibited a significant inhibition of intrahepatic tumor dissemination (Figure S7A). Furthermore, survival analysis demonstrated that the composite drug delivery system significantly prolonged the survival of tumor‐bearing mice (Figure 5J). Then, we harvested tumor tissues from each group of mice for histopathological examination. Notably, all treatment groups exhibited varying degrees of tumor necrosis, with the composite drug delivery system inducing the most extensive necrotic regions compared with other treatment modalities. Additionally, we found that in the mice treated with pachymaran‐loaded therapy, inflammatory cells were observed in the liver tissue adjacent to the tumor site (Figure 5K).

In addition, to verify whether the patch could inhibit tumor recurrence, we re‐established an orthotopic liver tumor recurrence model. After successful tumor establishment and growth to a predetermined size, we surgically removed tumors and applied the corresponding treatment to the resection site, then monitored tumor recurrence using in vivo live imaging (Figure 5L). As shown in Figure 5M, patch treatment significantly inhibited tumor recurrence and markedly prolonged mouse survival (Figure 5N). During treatment in the recurrence model, there was no significant difference in body weight changes between the two groups (Figure 5O). Collectively, these results indicate that the composite drug delivery system potentiates antitumor efficacy by effectively suppressing orthotopic tumor progression, distant metastasis, and postoperative recurrence.

To determine whether the therapeutic efficacy of the patch extends beyond localized PDT and involves systemic immune activation, a bilateral tumor model was established (Figure S8A). The composite patch was applied exclusively to the primary tumor, while the contralateral tumor remained untreated. Notably, treatment of the primary tumor resulted in a suppression of growth in the untreated distal tumors compared with control groups (Figure S8B–D). These findings demonstrate that localized patch therapy can elicit systemic antitumor effects, supporting the involvement of immune‐mediated mechanisms rather than solely localized PDT‐induced cytotoxicity.

### 2.7 MnO_2_‐P‐ICG NFs Promote Immune Cell Infiltration and Activation in Tumor Microenvironment

Preliminary cellular assays indicated that MnO_2_‐P‐ICG NFs reshaped the immune milieu and promoted NK cell activation. To substantiate this observation in vivo, we established an orthotopic liver cancer model according to the aforementioned protocol. To further clarify whether the composite drug delivery system could promote NK cell infiltration, we harvested tumor, spleen, and peritoneal lymph node tissues, which were subsequently processed for IHC staining using an anti‐CD49b antibody, a specific marker for NK cells. The results in Figure S7B demonstrated significantly increased NK cell infiltration in the tumor, spleen, and peritoneal lymph node tissues of the treatment groups, with the highest infiltration ratio observed in the composite drug delivery system treatment group, followed by the pachymaran‐loaded carrier group. These data suggest that our system effectively promotes NK cell infiltration into tumor tissues in mice, thereby favorably modulating the immune contexture of the tumor microenvironment.

**FIGURE 6:**
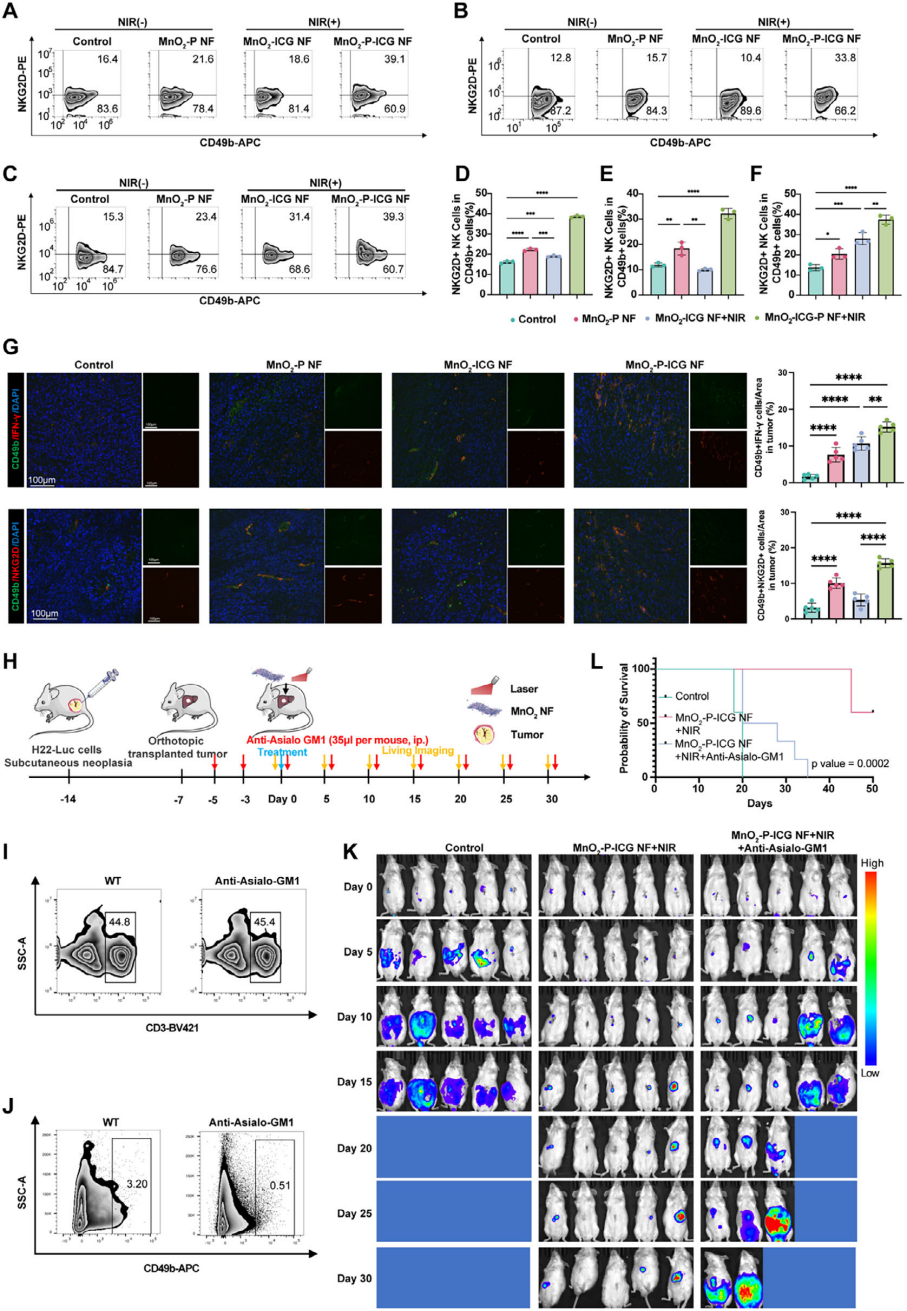
Enhanced NK cell activation contributes to antitumor efficacy and NK cell depletion impairs the therapeutic effect of the composite drug delivery system MnO_2_‐P‐ICG NFs. (A, D) Activation proportion of infiltrating NK cells in tumor tissues of each group of mice (*n *= 3/group). (B, E) Activation proportion of infiltrating NK cells in spleen tissues of each group of mice (*n *= 3/group). (C, F) Activation proportion of infiltrating NK cells in abdominal lymph node tissues of each group of mice (*n *= 3/group). (G) Representative immunofluorescence images of NKG2D and IFN‐γ expression, activation markers of NK cells, in mouse tumor tissues (scale bar = 100 µm, *n *= 5/group). (H) Schematic diagram of the in vivo antitumor therapy process. (I) Flow cytometry results show that NK cell depletion does not affect the proportion of T cells. (J) Flow cytometry results show that the anti‐Asialo‐GM1 antibody effectively depletes NK cells in mice. (K) In vivo fluorescence images showing the growth of orthotopic tumors in mice during treatment. (L) Survival status of mice in each group at day 50 post‐treatment (*n *= 5/group). Data were presented as mean ± SD. Ordinary one‐way ANOVA and log‐rank test were performed, *p < 0.05, **p < 0.01, ***p < 0.001, ****p < 0.0001.

The inhibition of NK cell activation within the tumor microenvironment poses a major challenge for solid tumor therapy. To verify whether our composite drug delivery system can improve the functional activation of NK cells in the tumor microenvironment, we assessed their activation ratio using flow cytometry. NK cells were identified as CD45^+^CD3‐CD49b^+^ cells within the gated population, and NKG2D^+^ NK cells were defined as activated NK cells. As shown in Figure 6A,D, the proportion of NK cells within the tumor tissues was higher in the treatment groups than in the control group, with the MnO_2_‐P‐ICG NFs group exhibiting the highest NK cell activation ratio. Moreover, the activation ratio of NK cells was higher in the MnO_2_‐P NF treatment group than in the MnO_2_‐ICG NF+NIR treatment group. These results indicate that our composite drug delivery system promotes NK cell activation within the tumor microenvironment, with pachymaran exerting a more pronounced activating effect on NK cells than the PDT effect mediated by ICG. Additionally, our composite system also facilitated NK cell activation in both the spleen (Figure 6B,E) and peritoneal lymph nodes (Figure 6C,F). Furthermore, immunofluorescence staining of mouse tumor tissues independently confirmed treatment‐induced NK cell activation. Compared to other groups, MnO_2_‐P‐ICG NFs significantly promoted the infiltration of CD49b^+^ NK cells, enhanced NKG2D expression on NK cells, and upregulated IFN‐γ expression (Figure 6G). Collectively, these results demonstrate that our composite drug delivery system can augment NK cell activation within the tumor microenvironment and favorably remodel the immune landscape in vivo, while simultaneously promoting NK cell activation in the spleen and lymph nodes.

In addition, we evaluated the effect of PDT on immunogenic cell death (ICD) using immunofluorescence analysis. PDT can induce ICD in cancer cells, leading to the release of tumor‐associated antigens and proinflammatory cytokines. The surface of dying cells typically undergoes characteristic alterations, including phosphatidylserine externalization and glycosylation changes, accompanied by the exposure of endoplasmic reticulum–resident proteins such as calreticulin (CRT) on the cell surface, which the immune system can recognize. ICD cells also typically release proinflammatory mediators, such as adenosine triphosphate (ATP) and high mobility group box‐1 protein (HMGB1), which serve to recruit and activate immune cells. These ICD‐associated damage‐associated molecular patterns (DAMPs) have been widely reported to function as innate immune stimulators, enhancing NK cell recruitment, activation, and cytotoxicity through stress‐sensing receptors and cytokine‐mediated signaling pathways. The results showed a marked increase in CRT and HMGB1 expression in the tumors of mice treated with PDT (Figure S9A,B). To quantitatively assess the induction of ICD, multiple canonical ICD‐associated markers were evaluated in vitro. Following treatment, flow cytometric analysis revealed enhanced cell surface exposure of CRT (Figure S10A,B). In parallel, ELISA measurements demonstrated elevated levels of HMGB1 release in the culture supernatants (Figure S10C). Consistently, a significant increase in extracellular ATP release was observed (Figure S10D). Together, these complementary indicators provide robust and quantitative evidence that the developed system effectively induces ICD in tumor cells. In summary, pachymaran more potently stimulated direct NK cell activation, whereas ICG further potentiated NK cell activation indirectly through ICD induction.

The aforementioned results demonstrate that our patch effectively activates NK cells to mediate antitumor activity. To verify the functional contribution of NK cells to this therapeutic effect, we systemically depleted NK cells in vivo. The experimental procedure is shown in Figure 6H. We used anti‐Asialo‐GM1 antibody to deplete NK cells in mice, and the depletion efficiency was validated by flow cytometry, which demonstrated effective NK cell depletion without significantly affecting the proportion of T cells (Figure 6I,J). By monitoring tumor growth and ascites metastasis in mice using live imaging, we found that NK cell depletion significantly attenuated the therapeutic efficacy of the patch (Figure 6K). Consistently, NK cell depletion significantly shortened mouse survival (Figure 6L). All these results indicate that NK cells are indispensable mediators of the antitumor efficacy of our therapeutic system.

### 2.8 MnO_2_‐P‐ICG NFs Activate the cGAS‐STING Pathway to Strengthen the Innate Immune Response

The cGAS‐STING pathway is a central innate immune signaling axis that senses cytosolic DNA, leading to immune cell activation and proinflammatory cytokine production, thereby establishing an effective innate immune defense against cancer cells [22]. Recent studies have shown that tumor‐derived DNA released into the tumor microenvironment can also stimulate the cGAS‐STING pathway. Accordingly, multiple studies have demonstrated that the antitumor efficacy of diverse therapeutic modalities relies, at least in part, on activation of the cGAS‐STING pathway [29, 30, 31, 32]. Given the ability of PDT‐induced ICD to promote cytosolic DNA accumulation and innate immune sensing, we next investigated whether MnO_2_‐P‐ICG NFs could activate the cGAS‐STING pathway.

We investigated whether the functional components of our composite drug delivery system could activate the cGAS‐STING pathway using Western blot (WB) analysis. As shown in Figure S11A–C, both ICG and MnO_2_ NF in the composite drug delivery system upregulated the expression of the downstream signaling proteins TBK1 and phosphorylated NF‐κB within the cGAS‐STING pathway in vitro. To further verify whether this system could promote immune activation and antitumor effects through activation of the cGAS‐STING pathway, we extracted proteins from tumor tissues in both the control and composite drug treatment groups for WB validation. As shown in Figure S11D–H, compared with the control group, the composite drug‐treated group markedly increased the expression of cGAS and phosphorylated STING in tumor tissues and enhanced the phosphorylation of downstream signaling molecules, including TBK1, NF‐κB, and IRF3. These results indicate that our composite drug delivery system enhances immune cell activation and innate immune responses, thereby potentiating antitumor efficacy through activation of the cGAS‐STING pathway.

To investigate the upstream events leading to activation of the cGAS‐STING pathway, DNA damage and cytosolic DNA accumulation were evaluated following treatment with the composite patch. A pronounced increase in cytoplasmic fragmented double‐stranded DNA (dsDNA) was observed in treated tumor cells (Figure S12A). In parallel, immunofluorescence analysis revealed markedly elevated γ‐H2AX expression, indicating enhanced DNA double‐strand break formation (Figure S12B). These findings provide direct evidence that the composite patch induces DNA damage and promotes cytosolic DNA accumulation, thereby supporting activation of the cGAS‐STING signaling pathway.

### 2.9 Inhibition of cGAS‐STING Pathway Weakens the Therapeutic Effect of MnO_2_‐P‐ICG NFs

To further elucidate the role of the cGAS‐STING pathway in our therapeutic system, we established a STING‐knockdown tumor cell line (Figure S13A,B). NK cells were co‐cultured with Hepa1‐6 tumor cells and subjected to the corresponding treatments. The activation status and cytotoxic activity of NK cells were assessed using flow cytometry. Our results demonstrated that inhibition of the cGAS‐STING pathway significantly reduced the proportion of activated NK cells and impaired their cytotoxic potential (Figure S13C–F). Additionally, to further investigate whether blockade of this pathway affects NK cell–mediated tumor cell killing, we conducted fluorescence staining assays. The findings revealed that inhibition of the cGAS‐STING pathway led to a marked reduction in tumor cell death (Figure S13G,H). Collectively, these results highlight the essential role of the cGAS‐STING pathway in mediating NK cell activation and cytotoxicity antitumor responses.

**FIGURE 7:**
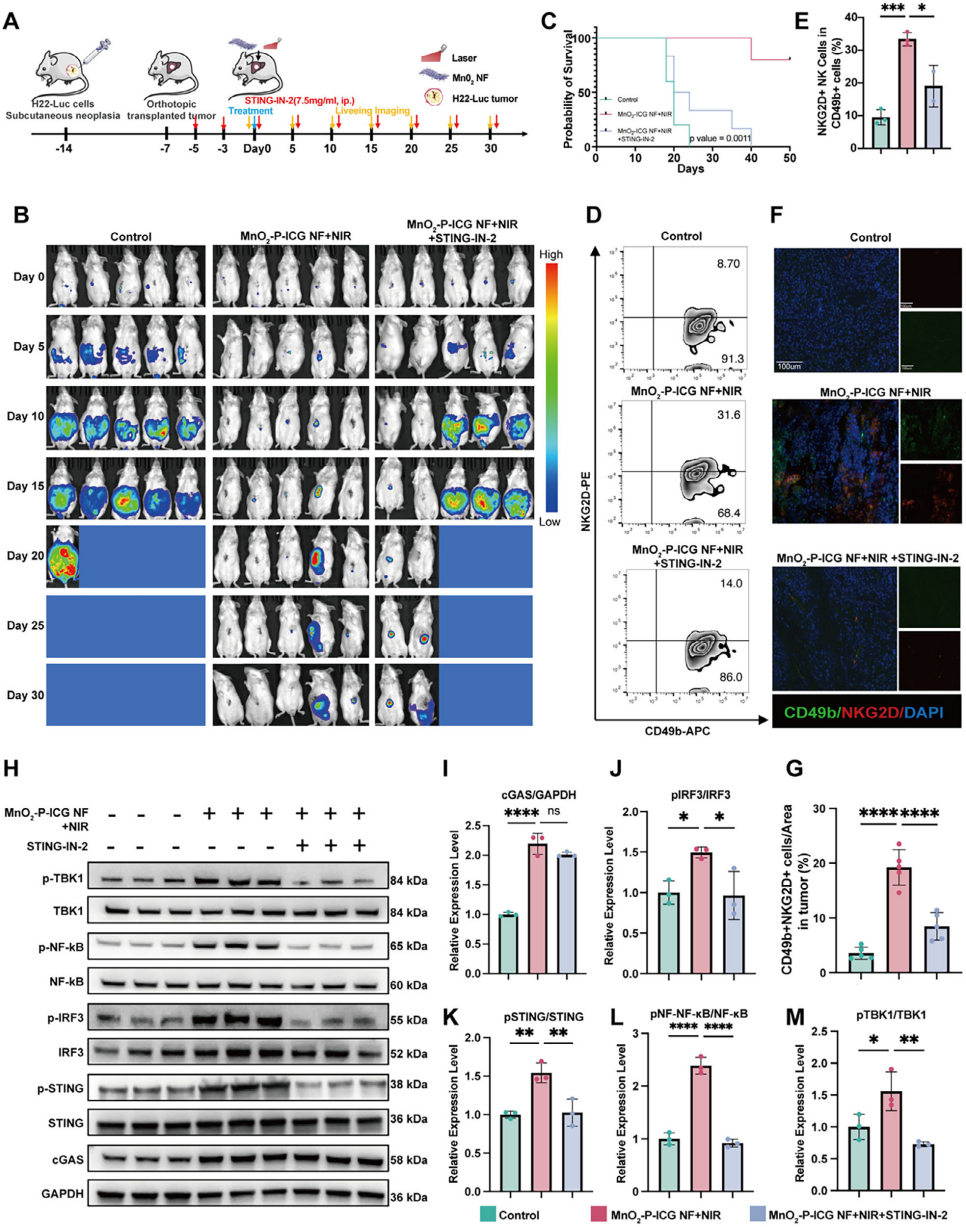
Inhibition of STING signaling impairs the therapeutic efficacy of the MnO_2_‐P‐ICG composite drug delivery system in orthotopic liver tumors. (A) Schematic diagram of the in vivo antitumor therapy process. (B) In vivo fluorescence images showing the growth of orthotopic tumors and ascites formation in mice during treatment. (C) Survival status of mice in each group at day 50 post‐treatment (*n *= 5/group). (D, E) Activation proportion of infiltrating NK cells in tumor tissues of each group of mice (*n *= 5/group). (F, G) Representative immunofluorescence images showing NKG2D expression, a marker of NK cell activation, in mouse tumor tissues (scale bar = 100 µm, *n* = 5/group). (H) Western blot results of the cGAS‐STING pathway proteins in the tumor tissues of the control group, composite drug treatment group and STING inhibitor groups. (I) Expression levels of cGAS in the tumor tissues (*n *= 3/group). (J) Expression levels of pIRF3/IRF3 in the tumor tissues (*n* = 3/group). (K) Expression levels of pSTING/STING in the tumor tissues (*n *= 3/group). (L) Expression levels of pNF‐κB/NFκB in the tumor tissues (*n *= 3/group). (M) Expression levels of pTBK1/TBK1 in the tumor tissues (*n *= 3/group). Data were presented as mean ± SD. Ordinary one‐way ANOVA and log‐rank test were performed, *p < 0.05, **p < 0.01, ***p < 0.001, ****p < 0.0001, ns = not significant.

To further investigate the role of the cGAS/STING pathway in mediating the therapeutic efficacy of our patch, we established an orthotopic liver cancer model in mice. We then administered a pharmacological STING inhibitor to specifically block the cGAS‐STING pathway and applied the corresponding treatments (Figure 7A). Tumor growth and metastasis were monitored using live imaging. The results revealed that pharmacological inhibition of the cGAS‐STING pathway significantly attenuated the therapeutic efficacy of our patch, particularly its ability to suppress tumor ascites metastasis (Figure 7B). Notably, cGAS‐STING pathway inhibition also significantly shortened mouse survival (Figure 7C). To elucidate the underlying mechanisms, we isolated primary tumor cells from each experimental group and performed flow cytometry analysis. The findings indicated that cGAS‐STING pathway inhibition reduced the proportion of activated NK cells (Figure 7D,E). Immunofluorescence staining further demonstrated a decreased proportion of CD49b^+^NKG2D^+^ NK cells within the tumor microenvironment (Figure 7F,G). Collectively, these observations demonstrate that cGAS‐STING pathway activity is required for patch‐mediated NK cell activation in vivo.

To further evaluate the impact of cGAS‐STING pathway inhibition on pathway activation, we performed WB analysis of tumor tissues to assess the expression of key signaling proteins. As shown in Figure 7H–M, pharmacological inhibition of the cGAS‐STING pathway significantly reduced the phosphorylation levels of TBK1, NF‐κB, and IRF3, thereby attenuating downstream signaling of the cGAS‐STING pathway. These results underscore the critical role of the cGAS‐STING pathway in mediating the therapeutic effects of our system through NK cell activation.

### 2.10 High Infiltration of NK Cells and Activated NK Cells Correlates With Improved Prognosis in HCC Patients

**FIGURE 8:**
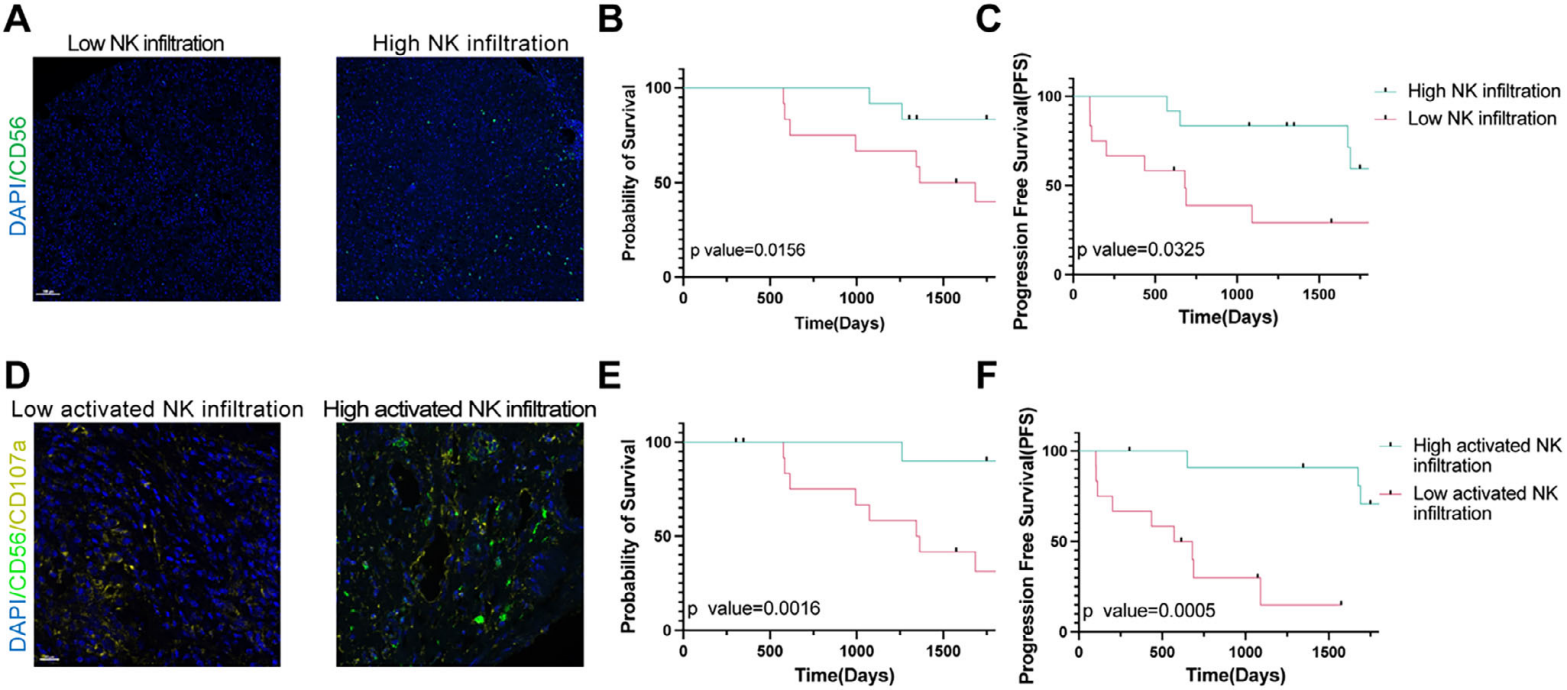
Intratumoral NK cell density and the proportion of activated NK cells in hepatocellular carcinoma (HCC) patients are associated with clinical outcomes. (A) Representative immunofluorescence images showing low and high infiltration of CD56^+^ cells in HCC tissues (Scale bar = 100 µm). (B, C) Kaplan‐Meier overall survival (OS) (B) and progression‐free survival (PFS) (C) curves of patients stratified according to CD56^+^ cell infiltration levels (*n *= 12/group). (D) Representative immunofluorescence images showing low and high infiltration of CD56^+^CD107a^+^ cells, representing activated NK cells, in HCC tissues (scale bar = 20 µm). (E, F) Kaplan‐Meier OS (E) and PFS (F) curves of patients stratified according to CD56^+^CD107a^+^ cell infiltration levels (*n *= 12/group). The log‐rank test was used for survival analysis.

To determine the prognostic significance of NK cell and activated NK cell infiltration in HCC, tumor specimens from 24 resected patients were analyzed by immunofluorescence staining, and clinical characteristics are summarized in Table S1. NK cells (CD56^+^) and activated NK cells (CD56^+^CD107a^+^) were quantified, and patients were dichotomized into high‐ and low‐infiltration groups using the median immunofluorescence score (*n* = 12/group). High NK cell infiltration was significantly associated with prolonged progression‐free survival (PFS) and overall survival (OS) compared with low infiltration (Figure 8A–C). Consistently, patients with high activated NK cell infiltration also exhibited markedly improved PFS and OS relative to the low‐infiltration group (Figure 8D–F). These findings highlight the favorable prognostic impact of both NK cell density and activation status in HCC, and further underscore the clinical translational potential of our therapeutic strategy. Notably, patients with high activated NK infiltration exhibited a lower incidence of microvascular invasion (MVI), particularly the absence of M2 grade invasion, compared with those with low activated NK infiltration (Table S1). In addition, highly activated NK infiltration was associated with a reduced frequency of satellite nodules and microscopic vascular tumor thrombus (Table S1), features that are commonly linked to early intrahepatic dissemination. When examined across pathological contexts associated with aggressive tumor behavior, including microvascular invasion status and tumor differentiation grade, these associations remained directionally consistent. Although these analyses are exploratory and limited by the modest sample size, the concordant trends observed across multiple high‐risk pathological subgroups suggest that activated NK cells may be involved in restraining vascular invasion and early metastatic spread in hepatocellular carcinoma.

## 3 Conclusion

HCC poses a significant threat to global health, ranking as the sixth most common cancer and the fourth leading cause of cancer‐related death worldwide. Primary liver cancer, predominantly HCC, is the leading cause of death among individuals under 65 years old in our country. Current therapeutic options for HCC are diverse, encompassing surgical intervention, interventional therapy, transplantation, targeted drug therapy, immunotherapy, and traditional Chinese medicine, among others [33]. Despite this diversity, effective prevention and treatment options are still scarce, resulting in high recurrence and mortality rates among patients with HCC. Enhancing the long‐term efficacy of frontline treatments and establishing novel therapeutic strategies are urgently needed. To counteract the hypoxic and immunosuppressive nature of the tumor microenvironment, we innovatively engineered hollow MnO_2_ NFs loaded with ICG and pachymaran via electrostatic adsorption for target tumor regions. This system gradually releases the loaded drugs and integrates PDT and immunomodulatory effects to enhance antitumor efficacy.

Due to the distances between some cancer cells and blood vessels exceeding the diffusion range of oxygen, hypoxia emerges as a prominent feature of the tumor microenvironment and is prevalent across various solid tumors [34]. Hypoxia exerts diverse effects throughout cancer progression, particularly by stimulating angiogenesis, fostering malignant cell proliferation, inhibiting apoptosis, and promoting immune evasion. Furthermore, the acidic environment resulting from extensive glycolysis in tumor cells further exacerbates immunosuppression and tumor malignancy. Metal ions play diverse roles in biological systems, serving as cofactors for enzymes and participating in cellular signaling [35]. These ions interact with immune sensors, ion transporters, enzymes, and downstream effector proteins through structural, catalytic, or regulatory mechanisms, thereby contributing to immune processes [36, 37]. Key metal ions such as K^+^, Ca^2+^, Mn^2+^, and Zn^2+^ play critical roles in regulating both innate and adaptive immunity [38, 39]. These metal ions can also act as sensitizers in immunotherapy, enhancing the immune system's responsiveness to treatment [40, 41]. Furthermore, metal complexes or metal ion inducers can trigger immunogenic cell death [42, 43, 44]. Targeting the hypoxic and acidic characteristics of the tumor microenvironment, we selected MnO_2_ as the foundational carrier to alleviate these unfavorable factors. By employing flexible silica/carbon composite fibers as templates and potassium permanganate as the manganese source, hollow MnO_2_ nanofibers were synthesized via hydrothermal reduction of potassium permanganate within the fibers. The hollow structure enhanced the surface area of the nanofibers, facilitating high loading of pachymaran and ICG. When the composite drug delivery system was localized to the tumor region, sustained and effective release of pachymaran could occur in the acidic environment. Additionally, MnO_2_ could react with H_2_O_2_ in the tumor microenvironment, generating O_2_ and Mn^2+^, alleviating tumor hypoxia, and providing oxygen for subsequent PDT. Moreover, Mn^2+^, as a metal ion, exhibited additional antitumor properties.

The liver is a reservoir for numerous immune cell types, including neutrophils, monocytes, Kupffer cells, NK cells, NKT cells, and lymphocytes. As a central metabolic organ, the hepatic environment exhibits high tolerance to pathogens, gut microbiota, and various metabolites to maintain overall homeostasis. This immunosuppressive polarization impairs T cell‐mediated antigen responses and instead relies on resident hepatic cell populations [3]. Most primary liver cancers develop under prolonged chronic inflammatory stimuli, during which tumor cells interact with immune cells and inflammatory factors within the immune microenvironment, creating a sanctuary for tumor cell formation and further inhibiting immune cell activation [5, 45]. Innate immune cells are integral to immune surveillance and antitumor responses, with NK cells, granular CD3‐ lymphocytes, capable of triggering innate immune responses against malignant tumor cells without the need for antigen presentation or complement activation. Increased cytotoxic activity of peripheral blood NK cells is positively correlated with a reduced cancer risk. In HCC, higher NK cell abundance signifies a better prognosis at early disease stages and correlates positively with tumor cell apoptosis. However, NK cells often lose their antitumor properties within the tumor microenvironment. Studies indicate a reduction in the frequency of peripheral blood and intrahepatic NK cells in HCC patients, with intratumoral NK cells expressing the inhibitory receptor NKG2A, which is associated with poor prognosis [5]. Cytokines within the tumor microenvironment interact with NK cells, thereby inhibiting their ability to infiltrate tumors and exert cytotoxic effects [46]. Pachymaran, a traditional antitumor Chinese medicine, has been shown in both domestic and international studies to possess antitumor effects and immunostimulatory properties. Our findings from intraperitoneal injection of pachymaran in a mouse model of liver cancer demonstrated enhanced infiltration of innate immune cells and activation of NK cells within tumor tissues after 2 weeks of treatment, as revealed by transcriptome sequencing. Sequencing data analysis revealed a role of pachymaran in promoting tumor infiltration by innate immune cells and activating NK cells. Our results demonstrated that pachymaran does not exert direct cytotoxic effects but instead exhibits host‐mediated antitumor properties. To address immune suppression and restricted innate immune responses within the tumor microenvironment, we loaded pachymaran and ICG onto MnO_2_ nanofibers via electrostatic adsorption. Upon localization to tumor sites, a gradual release occurs, promoting tumor cell apoptosis through PDT and activating immune responses within the tumor microenvironment. In vitro and in vivo experiments confirmed that pachymaran inhibited tumor cell migration, suppressed in vivo tumor metastasis, and enhanced NK cell‐mediated killing of tumor cells within the microenvironment. PDT within tumor regions promoted ICD and induced DNA release to activate the cGAS‐STING pathway. Mn^2+^ produced by the reaction of MnO_2_ with H_2_O_2_ within the tumor microenvironment further activated the cGAS‐STING pathway, promoting immune cell activation and improving the host immune status. It is important to note that ICD is characterized by the release of DAMPs, including ATP secretion, HMGB1 release, and calreticulin exposure. These ICD‐associated DAMPs have been widely reported to function as innate immune stimulators that enhance NK cell recruitment, activation, and cytotoxic function through stress‐sensing receptors and cytokine‐mediated signaling pathways. In this context, ICD induced by the composite drug delivery system may contribute to the establishment of a proinflammatory tumor microenvironment that favors NK cell activation. Consistent with this concept, activation of the cGAS–STING pathway observed in our study likely serves as a key innate immune signaling hub linking tumor cell stress and death to NK cell‐mediated antitumor immunity. Together, these findings provide a mechanistic rationale whereby ICD‐associated DAMPs and cGAS‐STING signaling cooperatively promote NK cell activation without necessitating direct involvement of adaptive immune components. It makes more sense that the proportion of NK cells infiltrated in liver cancer tissue was positively correlated with the good prognosis of patients.

In this study, our novel composite drug delivery system, MnO_2_‐P‐ICG NFs, demonstrated outstanding efficacy in suppressing tumor growth, metastasis, and recurrence, as well as synergistic immunotherapeutic effects against tumors. This work provides a feasible and effective strategy to enhance local tumor therapy. Firstly, our validation of the patch's ability to pass through a laparoscopic trocar highlights its flexibility, extensibility, and promising clinical application prospects. This method can be applied during minimally invasive surgeries or synchronously post‐hepatectomy. Notably, this study proposes an innovative strategy using a drug‐loaded patch that can be delivered through a laparoscopic trocar for local tumor therapy. Secondly, our patch is suitable for neoadjuvant or palliative treatment of large liver tumors protruding from the liver surface, as well as for patients at high risk of postoperative recurrence. It can generate immediate postoperative therapeutic effects on the resection surface, clearing scattered tumor cells and those with microvascular invasion. In addition, our drug‐loaded patch offers a therapeutic platform with potential for treating other intraperitoneal tumors, such as pancreatic, gastric, and colorectal cancers. Thirdly, current clinical treatments like TACE, microwave ablation, and radiotherapy are effective for local tumor therapy but cannot address potential micrometastases, thereby posing high recurrence risks. These treatments are often ineffective for multifocal tumors and require a high level of operator skills. Improper operation may lead to incomplete tumor eradication. In contrast, our patch, when used in laparoscopic procedures, is relatively simple and intuitive. It can be directly applied under laparoscopy following hepatectomy. It can not only induce local tumor cell death but also recruit and activate surrounding NK cells to eliminate tumor cells, target microvascular infiltration and scattered tumor cells, and inhibit micrometastases. For intrahepatic multifocal cases, the patch size can be tailored according to tumor location and size, offering an alternative or combinational therapeutic option.

In conclusion, this study established a composite drug delivery system, termed MnO2‐P‐ICG NFs, loaded with pachymaran and ICG for targeted tumor immunotherapy and PDT. Both in vitro and in vivo experiments demonstrated the excellent biocompatibility of the composite drug delivery system. Under the acidic conditions of the tumor microenvironment, it exhibited acid‐responsive behavior and facilitated the sustained release of pachymaran. Primarily, through transcriptomic analysis of tumor tissues and flow cytometry analysis of primary cells after pachymaran treatment, we observed that pachymaran promoted the infiltration of innate immune cells into tumors and activated NK cells in vivo. In vitro experiments revealed that the composite drug delivery system inhibited tumor cell proliferation and migration while also promoting NK cell activation and enhancing their cytotoxicity against tumor cells. In vivo experiments further demonstrated that the composite drug delivery system suppressed the growth of primary tumors, inhibited tumor metastasis to the peritoneal cavity and liver, reduced tumor recurrence and extended survival. Subsequent flow cytometry and immunohistochemistry experiments indicated that our composite drug delivery system enhanced the infiltration of NK cells into the tumor microenvironment and boosted their cytotoxic activity. Depletion of NK cells in mice compromised the therapeutic efficacy of this system. Mechanistically, we found that the composite drug delivery system improved the tumor immune microenvironment by inducing ICD‐associated stress responses, leading to the release of DAMPs and subsequent activation of the cGAS‐STING pathway, which in turn promoted NK cell‐mediated innate immune activation. Pharmacological inhibition of this pathway significantly attenuated the therapeutic efficacy of the system. Consequently, our developed composite drug delivery system holds significant implications for the treatment of malignant liver tumors. Based on nanomaterials and traditional herbal medicine, we propose a biocompatible strategy that effectively integrates PDT and immunotherapy, demonstrating substantial potential as a novel therapeutic approach for malignant liver tumors.

While this study provides valuable insights, several limitations warrant further investigation. Firstly, although our findings demonstrate that the composite drug delivery system promotes immune cells infiltration into tumors through activation of the cGAS‐STING pathway, the mechanism by which pachymaran activates innate immune cells requires further investigation to facilitate subsequent clinical translation. Second, while this study focuses on NK cell‐mediated innate immunity, the involvement of other immune cell subsets, as well as potential crosstalk between innate and adaptive immunity, was not systematically examined. Future studies incorporating more comprehensive immune profiling will be necessary to elucidate these interactions further and fully define the immunological mechanisms underlying the observed therapeutic effects.

## 4 Experimental Methods

### 4.1 Ethics Approval

This study involving human participants was approved by the Clinical Research Ethics Committee of the Mengchao Hepatobiliary Hospital of Fujian Medical University (Approval No. 2021_100_06). Written informed consent was obtained from all participants. Sex and/or gender were determined based on self‐report. All animal experiments were approved by the Animal Experimental Ethical Inspection of Zhejiang University (Approval No. ZJU20230500).

### 4.2 Cell Culture

This study involves three cell lines. The mouse hepatoma cell line Hepa1‐6 (No. TCM39, RRID: CVCL_0327) and the mouse normal liver cell line AML12 (No. GNM42, RRID: CVCL_0140) were purchased from the Cell Bank of the Chinese Academy of Sciences. The mouse hepatoma cell line H22 (No. CL‐0341, RRID: CVCL_H613) was purchased from Procell Life Science & Technology Co., Ltd. Hepa1‐6 and AML12 cells were cultured in Dulbecco's Modified Eagle's Medium (DMEM) supplemented with 10% fetal bovine serum (FBS) and 1% penicillin‐streptomycin, whereas H22 cells were cultured in Roswell Park Memorial Institute (RPMI) 1640 medium containing 10% FBS and 1% penicillin‐streptomycin. Primary immune cells were isolated from mouse spleens, and primary NK cells (CD45^+^ CD3‐ CD49b^+^) were sorted by flow cytometry and cultured in NK cell‐specific medium (No. iCell‐i006‐002m, Cellverse Co., Ltd.). All cells were maintained at 37°C in a humidified incubator with 5% CO_2_. Mycoplasma contamination was routinely tested every 4 weeks using the PCR‐based Mycoplasma Detection Kit (No. HY‐K0552, MedChemExpress). The absence of mycoplasma contamination in all cultured cells was confirmed before experimentation. The most recent mycoplasma test results are provided in Figure S14.

### 4.3 Materials

Tetraethyl orthosilicate, phosphoric acid, polyvinyl alcohol, potassium permanganate, polyethyleneimine (PEI), and indocyanine green (ICG) were purchased from Sigma‐Aldrich (Beijing, China). Pachymaran was purchased from Winherb (Shanghai, China). RPMI 1640 medium, DMEM, trypsin, penicillin‐streptomycin, and FBS were purchased from Gibco (Burlington, USA). The CFSE Cell Proliferation Kit was purchased from Thermo Fisher Scientific (Burlington, USA). Primary antibodies against cGAS, phospho‐STING, STING, GAPDH, NF‐κB p65, phospho‐NF‐κB p65, IRF3, phospho‐IRF3, TBK1/NAK, and phospho‐TBK1/NAK were purchased from Cell Signaling Technology (Massachusetts, USA). Fluorochrome‐conjugated antibodies against mouse CD3, CD45, CD49b, NKG2D, and NK1.1 were purchased from BioLegend (California, USA). STING‐IN‐2 (HY‐138682) was purchased from MedChemExpress (Shanghai, China). All chemicals were used as received without further purification. Detailed information regarding the antibodies is systematically summarized in Table S2.

### 4.4 Construction of the Composite Drug Delivery System

Silica gel fibers were prepared via the sol‐gel method, with tetraethyl orthosilicate as the precursor, deionized water as the solvent, phosphoric acid as the catalyst, and polyvinyl alcohol as the spinning aid. By adjusting the ratio of deionized water, hydrolysis time, and hydrolysis temperature, precursor solutions with different viscosities, surface tensions, and conductivities were obtained. Key parameters for achieving stable electrospinning, including the applied voltage range and flow rate range, were investigated, resulting in silica gel fibers with diameters ranging from 100 to 300 nm. The as‐prepared silica gel fibers were subsequently sintered in a nitrogen atmosphere to obtain silica/carbon composite fibers. The effects of sintering temperature and duration on the morphology, size, structure, and flexibility of the fibers were systematically evaluated. Using the obtained flexible silica/carbon composite fibers as templates, hollow manganese dioxide (MnO_2_) nanofibers were synthesized via hydrothermal reduction of potassium permanganate by the carbon component of the fibers. The effects of the fiber‐to‐potassium permanganate ratio, hydrothermal temperature, and reaction time on the morphology and size of the nanofibers were systematically investigated, providing a theoretical basis for the synthesis of composite nanofibers using flexible silica fiber templates. The hollow MnO_2_ nanofibers were surface‐modified with PEI by stirring in a PEI aqueous solution. After modification, the nanofibers were incubated with an aqueous solution containing pachymaran and ICG, enabling their loading onto the fibers via electrostatic adsorption. The final product, designated as MnO_2_‐P‐ICG nanofibers (with MnO_2_‐P NF and MnO_2_‐ICG NF prepared using the same procedure), was obtained.

### 4.5 UV–Vis Spectroscopy

The absorbance of ICG and pachymaran at different concentrations was measured using UV–vis spectroscopy. Standard calibration curves for ICG and pachymaran were obtained to calculate their loading capacity and loading efficiency.

### 4.6 Drug Release Analysis

MnO_2_‐ICG NF and MnO_2_‐P NF were dispersed in solutions with different pH values (pH = 5.0, 6.0, 6.8, 7.2). At predetermined time points, the supernatant was collected, and the released ICG and pachymaran were quantified using UV–vis spectroscopy. An equal volume of fresh solution with the same pH was added to maintain constant release conditions. The cumulative release profiles of ICG and pachymaran were subsequently evaluated.

### 4.7 Oxygen Generation Performance

Changes in dissolved oxygen content in MnO_2_‐P‐ICG NFs solution were measured using an oxygen meter under acidic conditions (pH = 5.8) in the presence of H_2_O_2_ to evaluate its oxygen‐generating capability.

### 4.8 In Vitro PDT Performance

The in vitro photodynamic therapy (PDT) performance was evaluated using 1,3‐diphenylisobenzofuran (DPBF). MnO_2_‐ICG‐P NF was added to the DPBF solution, which was then divided into four groups according to the presence or absence of H_2_O_2_ and NIR irradiation. The solution pH was adjusted to 5.8, and the changes in absorbance at 411 nm were monitored.

### 4.9 Free Radical Detection

The types of free radicals generated were detected using electron spin resonance (ESR) spectroscopy with 2,2,6,6‐tetramethylpiperidine (TEMP) employed as a spin‐trapping agent for singlet oxygen (^1^O_2_). Under acidic conditions (pH = 5.8), two experimental groups were established: H_2_O_2_ + TEMP + MnO_2_‐ICG‐P NF with NIR and a corresponding control group. The ESR spectra were recorded at room temperature.

### 4.10 Cellular Uptake

Hepa1‐6 cells (5 × 10^4^ cells/mL) were seeded in 24‐well culture plates containing 500 µL of complete medium per well and incubated overnight, after which MnO_2_ NF@FITC (0.1 mg/mL) was added. Cellular uptake was monitored at different time points using a fluorescence microscope (EVOS FL Auto 2, Thermo Fisher Scientific, USA).

### 4.11 Cryo‐TEM Cell Imaging

Cells were pretreated with MnO_2_ NFs for the indicated durations in a 37°C incubator. The cell suspension was transferred to a clean 1.5 mL conical EP tube and centrifuged at 1000 rpm for 5–10 min. After discarding the supernatant, the cell pellet was retained. Subsequently, 2.5% (v/v) glutaraldehyde fixative (Aladdin, Cat. No. 111308) was gently added to the pellet. Using a toothpick, the pellet was gently loosened to prevent compact sedimentation and fixed at room temperature for 3–5 min under light‐protected conditions, followed by storage at 4°C overnight. Finally, samples were processed and stained at a cryo‐electron microscopy core facility and imaged using a TEM (Thermo Scientific Talos L120C).

### 4.12 In Vitro Photodynamic Therapy

Hepa1‐6 cells (5 × 10^4^ cells/mL) were evenly seeded in well plates and cultured for 24 h. Each group was then treated with P, ICG, MnO_2_ NF, and MnO_2_‐P‐ICG NFs at a concentration of 100 µg/mL, respectively. After incubation for 6 h, the cells were irradiated with a continuous‐wave 808 nm laser at 1 W/cm^2^ for 120 s. Following a further 24 h incubation in complete culture medium, CCK‐8 assays, protein extraction, and immunofluorescence analyses were subsequently performed.

### 4.13 In Vitro Immune Co‐Culture

Hepa1‐6 cells (5 × 10^4^ cells/mL) were evenly seeded in a 12‐well plate and cultured for 24 h. The medium was discarded, and the cells were washed three times with PBS. The Hepa1‐6 cells were then labeled with carboxyfluorescein diacetate succinimidyl ester (CFSE) and washed three times with PBS. Each group was treated with P, ICG, MnO_2_ NFs, or MnO_2_‐P‐ICG NFs at a concentration of 100 µg/mL, respectively. After 6 h, the cells were irradiated with a continuous‐wave 808 nm laser at 1 W/cm^2^ for 120 s. Primary immune cells were isolated from mouse spleens, and primary NK cells (CD45^+^ CD3‐ CD49b^+^) were sorted by flow cytometry at a density of 50 × 10^4^ cells/mL. These NK cells were co‐cultured with Hepa1‐6 cells in the well plates for 8 h.

### 4.14 Cell Transfection

siRNA targeting STING (si‐STING) and negative control siRNA (si‐NC) were synthesized by Tsingke Biotechnology Co., Ltd. (Beijing, China). These oligonucleotides (5 µL of 100 nm si‐NC or si‐STING) were transfected into cells using Lipofectamine 3000 reagent (Invitrogen, Carlsbad, CA, USA), according to the manufacturer's instructions. The siRNA sequences for si‐STING and si‐NC (5′–3′) were as follows: si‐STING: 5’‐GCAUCAAGGAUCGGGUUUA‐3’ (forward) and 5’‐UAAACCCGAUCCUUGAUGCTT‐3’ (reverse); si‐NC: 5’‐UUCUCCGAACGUCACGUTT‐3’ (forward) and 5’‐ACGUGACACGUUCGGAGAATT‐3’ (reverse).

### 4.15 Construction of a Mouse Subcutaneous Tumor Model

Four‐week‐old male BALB/c or BALB/c nude mice were selected and acclimated for one week in the animal experimental center. After acclimation, 1 × 10^6^ H22‐Luc cells (for BALB/c mice) or Hepa1‐6‐Luc cells (for BALB/c nude mice) were subcutaneously injected into both inguinal regions of the mice. The tumor cell suspension was mixed with Matrigel at a 1:1 (v/v) ratio. Following the injection, the injection sites were gently pressed for several seconds using a sterile cotton swab moistened with PBS. The mice were then maintained under standard housing conditions for two weeks, during which subcutaneous solid tumors with a size of approximately 0.5 × 0.5 × 0.5 cm^3^ formed in the inguinal regions.

Before orthotopic tumor implantation, tumor‐bearing donor mice were euthanized. Subcutaneous tumor tissues were excised and isolated using ophthalmic scissors, and necrotic or hemorrhagic regions were removed. The viable tumor tissues were then cut into small fragments of approximately 1 mm × 1 mm × 1 mm (approximately 1 mg) and placed in sterile PBS.

### 4.16 Construction of an Orthotopic Liver Cancer Mouse Model

The mouse was anesthetized using an inhalation anesthesia system for small animals and securely positioned on a specialized mouse surgical board. A midline abdominal incision, approximately 1 cm in length, was made below the xiphoid process using ophthalmic scissors. The tissues were dissected layer by layer until the liver was exposed. Using a sterile cotton swab moistened with PBS, the left lateral lobe of the liver was gently externalized by applying slight pressure. The previously prepared tumor fragments (1 mm^3^) were then grasped with ophthalmic forceps, with the tips oriented downward, and slowly inserted into the liver to a depth of approximately 1–2 mm. The tumor tissue was gently pushed into the liver parenchyma, and the forceps were carefully withdrawn. The position of the tumor tissue was adjusted using forceps to ensure that it remained in place. Hemostasis was achieved by gently pressing bleeding sites on the liver with a sterile PBS‐moistened cotton swab until bleeding ceased. The liver was then returned to the abdominal cavity. The abdominal cavity was closed in layers using 5‐0 sutures. The mouse was placed on an electric heating pad to maintain body temperature and monitored until it regained consciousness, at which point it was transferred to a housing cage.

### 4.17 Construction of a Tumor Recurrence Mouse Model

The animal experiments were performed in accordance with a previously reported protocol [47]. Briefly, an orthotopic liver cancer model was first established as described above. One week after tumor implantation, the mice were randomly assigned to different groups. Following anesthesia, the primary tumors were surgically resected, after which the corresponding treatments were administered. Two weeks after surgery, live imaging was performed to monitor tumor recurrence and distant metastasis.

### 4.18 In Vivo Imaging of Orthotopic Tumors

Approximately one week after orthotopic tumor implantation, tumor bioluminescence signals were detected using an IVIS in vivo imaging system. Starting one week post‐implantation, tumor bioluminescence signals were monitored and recorded every five days. Before each IVIS imaging session, mice were intraperitoneally injected with a luciferase substrate (D‐luciferin) at a dose of 10 µL/g body weight. After the injection, the mice were placed in an anesthesia induction chamber. Approximately 10–15 min later, the mice were transferred to the IVIS system for image acquisition of tumor bioluminescence signals.

### 4.19 NK Cell Depletion

For NK cell depletion, mice were intraperitoneally (i.p.) injected with anti–asialo GM1 antibody (Poly21460, BioLegend, 35 µL per mouse) 5 and 3 days before, and 1 day after treatment. Antibody injections were subsequently performed every 5 days throughout the course of the experiment.

### 4.20 In Vivo Targeted Tumor Therapy

Five days after the establishment of orthotopic tumors, tumor bioluminescence signals were verified using an IVIS in vivo imaging system. The mice were then randomly divided into four groups: Control, MnO_2_‐P NFs, MnO_2_‐ICG NFs, and MnO_2_‐P‐ICG NFs. After an 8 h fasting period, the mice were anesthetized using an inhalation anesthesia system and securely fixed on a specialized surgical board. A midline abdominal incision of approximately 1 cm was made below the xiphoid process using ophthalmic scissors. The abdominal tissues were dissected layer by layer until the liver was exposed. Using a sterile cotton swab moistened with PBS, the left lateral lobe of the liver was gently externalized by applying slight pressure. The orthotopic tumor was identified, and 100 µg of drug‐loaded fibrous material was applied directly to the tumor surface. The tumor was then irradiated with a continuous‐wave 808 nm laser at 1 W/cm^2^ for 10 min. After irradiation, the liver was carefully returned to the abdominal cavity, and the incision was closed in layers using 5‐0 sutures. The treated mice were placed on an electric heating pad to maintain body temperature and closely monitored until they regained consciousness. Once awake, they were returned to their housing cages and maintained under standard conditions.

### 4.21 Collection of Mouse Mesenteric Lymph Nodes

Following euthanasia, the abdominal cavity was carefully exposed to locate and harvest mesenteric lymph nodes as follows: (1) Identification: The cecum was first identified, and the mesenteric attachment was traced proximally toward the intestinal convergence point. (2) Exposure: The gastrointestinal tract was gently repositioned to reveal the gastroduodenal junction. (3) The mesentery associated with the small intestine was visually identified. Mesenteric lymph nodes appeared as milky‐white to pale yellow structures, denser than surrounding adipose tissue, and typically exhibited a white, elongated morphology. (4) Harvesting: The mesenteric tissue was carefully harvested and processed for cell separation experiments

### 4.22 Flow Cytometry for Apoptosis Detection

Collected cells were washed twice with pre‐cooled PBS and centrifuged at 300 × g for 5 min at 4°C. Approximately 1–5 × 10^5^ cells were collected. The supernatant was removed, and the cells were resuspended in 500 µL of 1× Binding Buffer. Then, 5 µL of Annexin V‐FITC and 10 µL of PI were added to the cell suspension and gently mixed. The mixture was incubated at room temperature in the dark for 15 min. The flow cytometer parameters were adjusted, and the samples were analyzed.

### 4.23 Flow Cytometry for NK Cell Activation

Collected cells were washed twice with pre‐cooled PBS and centrifuged at 300 × g for 5 min at 4°C. Approximately 1–5 × 10^5^ cells were collected. The supernatant was discarded, and the cells were resuspended in 100 µL of cell staining buffer. An appropriate amount of Fc receptor blocking solution was added to the cell suspension, mixed, and incubated on ice in the dark for 5–10 min. The required fluorescent antibodies were then added at the predetermined optimal concentrations and incubated on ice in the dark for 15–20 min. The cells were washed twice with cell staining buffer, centrifuged at 300 × g for 5 min at 4°C, resuspended, and 5 µL of 7‐AAD viability staining solution was added. The mixture was incubated on ice in the dark for 5 min, and then the proportions of target cells were detected using a flow cytometer.

### 4.24 Western Blotting

Tumor tissues were homogenized for total protein extraction. Proteins were separated by sodium dodecyl sulfate–polyacrylamide gel electrophoresis (SDS–PAGE) and transferred onto membranes for immunoblotting, followed by detection using an enhanced electrochemiluminescence (ECL) reagent. The following primary antibodies were used: rabbit anti‐cGAS (Cell Signaling Technology, Cat# 79978T), rabbit anti‐phospho‐STING (Cell Signaling Technology, Cat# 19781T), rabbit anti‐STING (Cell Signaling Technology, Cat# 13647T), mouse anti‐GAPDH (Cell Signaling Technology, Cat# 97166T), rabbit anti‐NF‐κB p65 (Cell Signaling Technology, Cat# 8242T), rabbit anti‐phospho‐NF‐κB p65 (Cell Signaling Technology, Cat# 3033T), rabbit anti‐IRF3 (Cell Signaling Technology, Cat# 4302S), rabbit anti‐phospho‐IRF3 (Cell Signaling Technology, Cat# 4947S), rabbit anti‐TBK1/NAK (Cell Signaling Technology, Cat# 3504T), rabbit anti‐phospho‐TBK1/NAK (Cell Signaling Technology, Cat# 5483T), rabbit anti‐NKG2D (Abcam, Cat# ab203353), rabbit anti‐interferon‐γ (IFN‐γ) (Abcam, Cat# ab231036), rabbit anti‐integrin α2 (Abcam, Cat# ab181548), rabbit anti‐HMGB1 (Abcam, Cat# ab18256), and rabbit anti‐calreticulin (Abcam, Cat# ab92516).

### 4.25 Statistical Analysis

In the experiments described above, each group or condition included at least three independent biological replicates. Descriptive statistics for quantitative data were presented as mean ± standard deviation (SD). Sample sizes (n) were indicated in each figure legend. Appropriate statistical analysis methods were selected based on the data type. Comparisons between two groups were conducted using Student's t‐test, whereas comparisons among multiple groups were analyzed using one‐way ANOVA or two‐way ANOVA. Survival curve analysis was performed using the log‐rank test. A *p* value < 0.05 was considered statistically significant. All statistical analyses were performed using GraphPad Prism version 10.1.1 (GraphPad Software).

## Author Contributions

J.L, H.P, K.W contributed equally to this work. J.L, D.C, X.C, J.Y. conceived the work, performed the experiments, analyzed the data and wrote the paper. K.W, J.N, J.L. contributed to the MnO_2_‐P‐ICG NFs characterization and flow cytometry assays. J.D, Y.C, J Y, H.L, B.L. contributed to the animal experiments. Y.S, H.S, Q.Y, M.W. conceived and supervised the project and wrote the paper.

## Funding

This work was supported by the funding from Key Research and Development Project of Zhejiang Province (2021C03061) and Zhejiang Clinical Research Center of Minimally Invasive Diagnosis and Treatment of Abdominal Diseases (2018E50003); the National Natural Science Foundation of China (82102903) and Zhejiang Medical Health Science and Technology Program (2023RC031); Natural Science Foundation of Zhejiang Province (LR25C100002); the Postdoctoral Fellowship Program of CPSF (GZB20250520).

## Conflicts of Interest

The authors declare no conflicts of interest.

## Supporting information




**Supporting File 1**: advs75009‐sup‐0001‐SuppMat.pdf.

**Supporting File 2**: advs75009‐sup‐0002‐DataFile.txt.

**Supporting File 3**: advs75009‐sup‐0003.csv.

## Data Availability

The data that support the findings of this study are available in the supplementary material of this article.
