# Supplementary material for: Laparoscopic Delivery of a MnO2‐P‐ICG Patch for Photodynamic Therapy and NK Cell‐Driven Immunotherapy in Hepatocellular Carcinoma
Source: Adv Sci (Weinh). 2026 Apr 3;13(32):e24246. doi: 10.1002/advs.202524246 (PMC13252648; doi:10.1002/advs.202524246)
Supplement: Supplementary file 1 — Supporting File 1: advs75009‐sup‐0001‐SuppMat.pdf. [file ADVS-13-e24246-s003.pdf]

Supporting Information

Laparoscopic Delivery of a Versatile Patch for NK Cell-Driven Immunotherapy Against Hepatocellular Carcinoma

Jie Lin, Haoqi Pan, Ke Wu, Junjie Nan, Jinyao Dai, Yushun Chang, Hao Shen, Qingxuan Ye, Haowen Lu, Yuxuan Shen, Boqiang Liu, Ming Wu, Jicheng Yu*, Xiujun Cai*, Dong Cen**

This PDF file includes:

Figs. S1 to S25
Data S1 to S2
Table S1 to S2

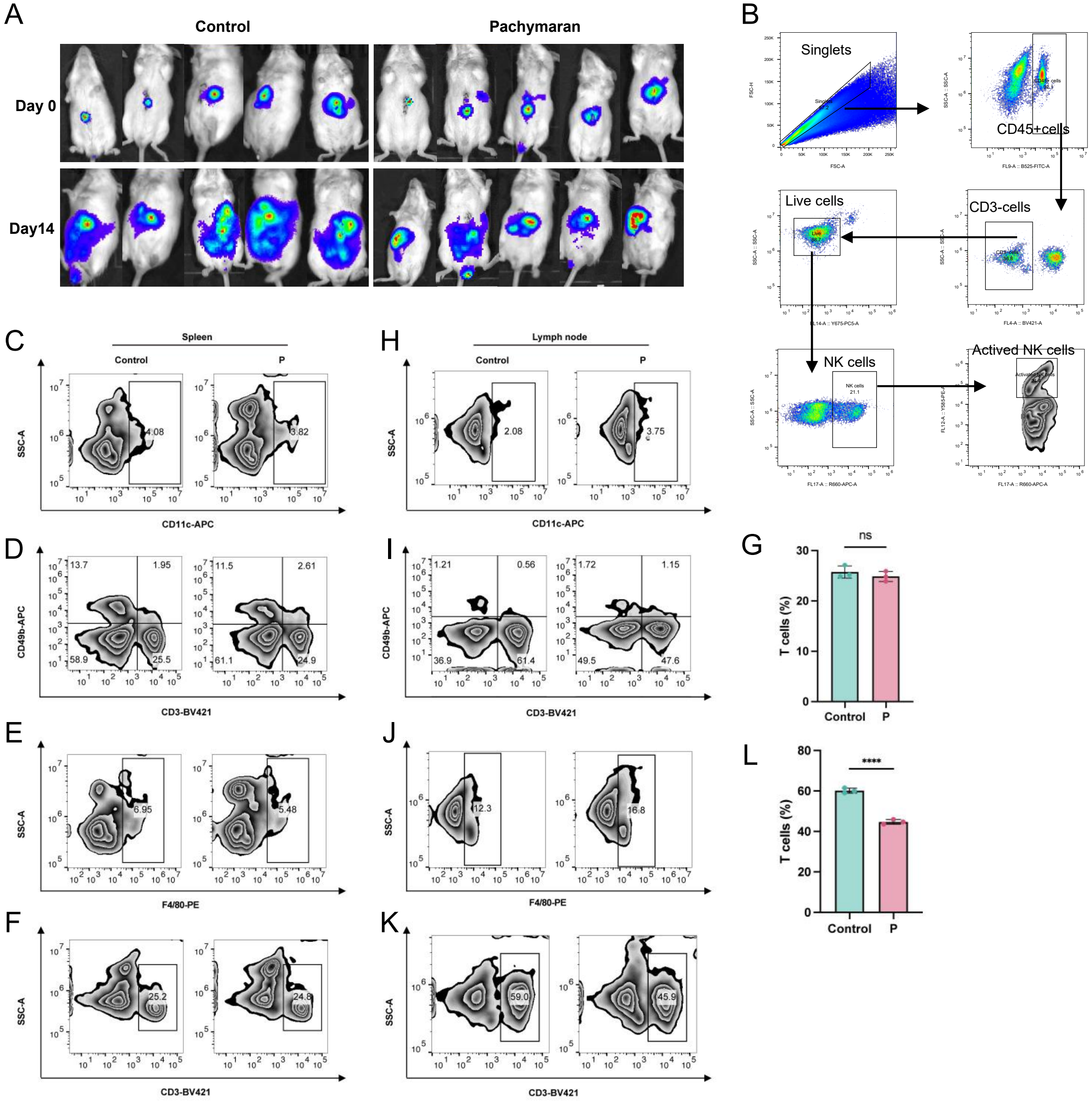

Figure S1. Regulation of the immune microenvironment by pachymaran.

(**A**) The therapeutic effects of pachymaran on H22 orthotopic liver tumors. (**B**) Flow cytometry gating strategy. (**C-G**) Effects of pachymaran on DCs (**C**), NK cells (**D**), macrophages (**E**) and T cells (**F, G**) in the spleen of mice (*n* = 3/group). (**H-L**) Effects of pachymaran on DCs (**H**), NK cells (**I**), macrophages (**J**) and T cells (**K, L**) in the peritoneal lymph nodes of mice (*n* = 3/group). Data were presented as mean ± SD. Unpaired t-test was performed, ****$p < 0.0001$, ns = not significant.

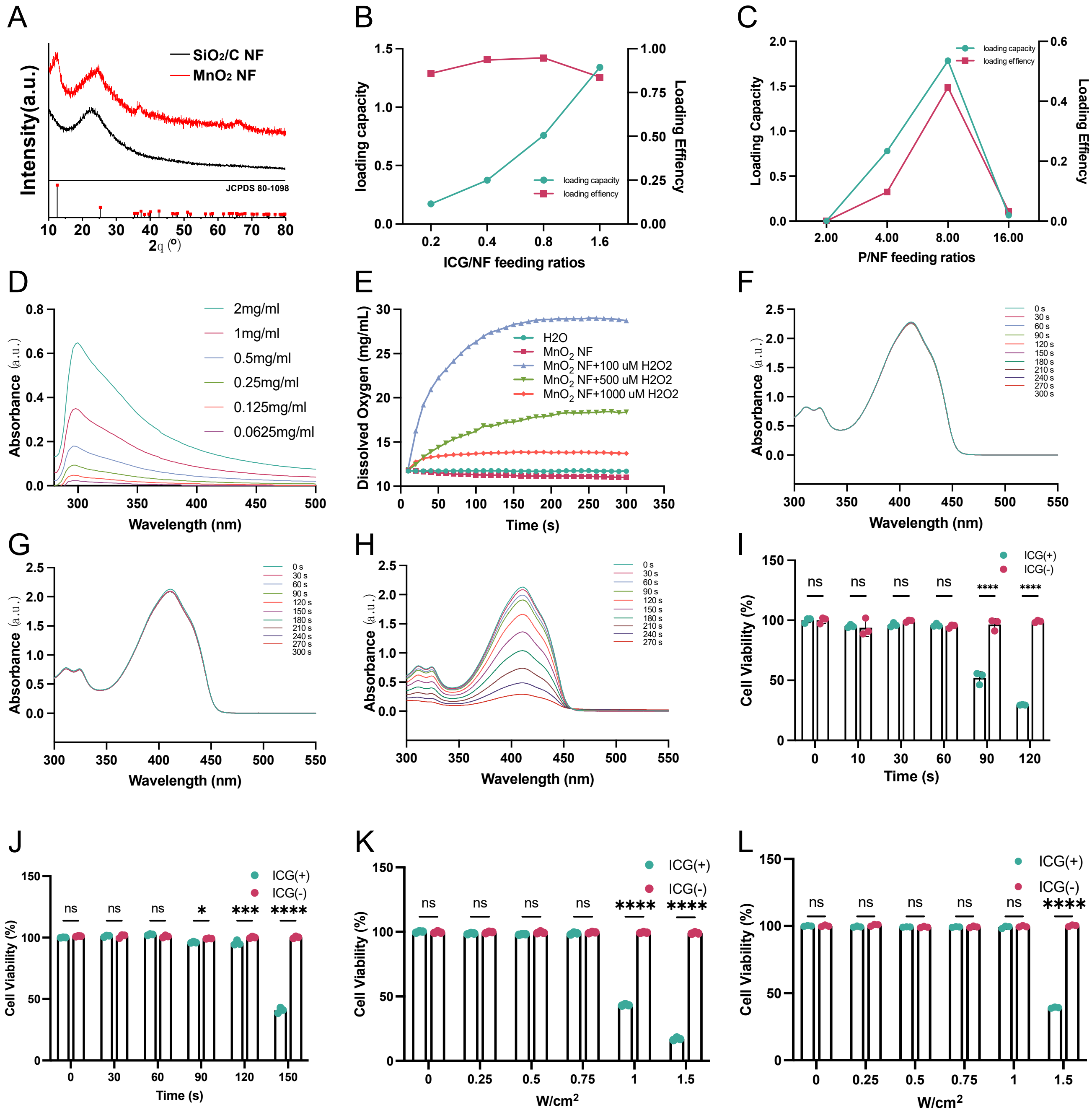

Figure S2. Characterization of functional component loading in the MnO_2-P-ICG NFs composite drug delivery system.

(**A**) XRD phase analysis of SiO_2/C nanofibers and hollow MnO_2 NFs. (**B, C**) Detection of loading capacity and efficiency for pachymaran and ICG. (**D**) UV-vis spectroscopy for detecting pachymaran. (**E**) Oxygen generation capability of MnO_2-P-ICG NFs measured using a dissolved oxygen meter. (**F-H**) Degradation rate of DPBF over time under 808 nm NIR (**F**), under 808 nm

NIR with MnO_2-P-ICG NFs (**G**), and under 808 nm NIR with the presence of H_2O_2 and MnO_2-P-ICG NFs (**H**). (**I**) Effects of 808 nm NIR on the growth and proliferation of mouse liver cancer cells at different time points (n = 3/group). (**J**) Effects of 808 nm NIR on the growth and proliferation of mouse immune cells at different time points (n = 3/group). (**K**) Effects of 808 nm NIR on the growth and proliferation of mouse liver cancer cells at different doses (n = 3/group). (**L**) Effects of 808 nm NIR on the growth and proliferation of mouse immune cells at different doses (n = 3/group). Data were presented as mean ± SD. Two-way ANOVA was performed, $*p < 0.05$, $***p < 0.001$, $****p < 0.0001$, ns = not significant.

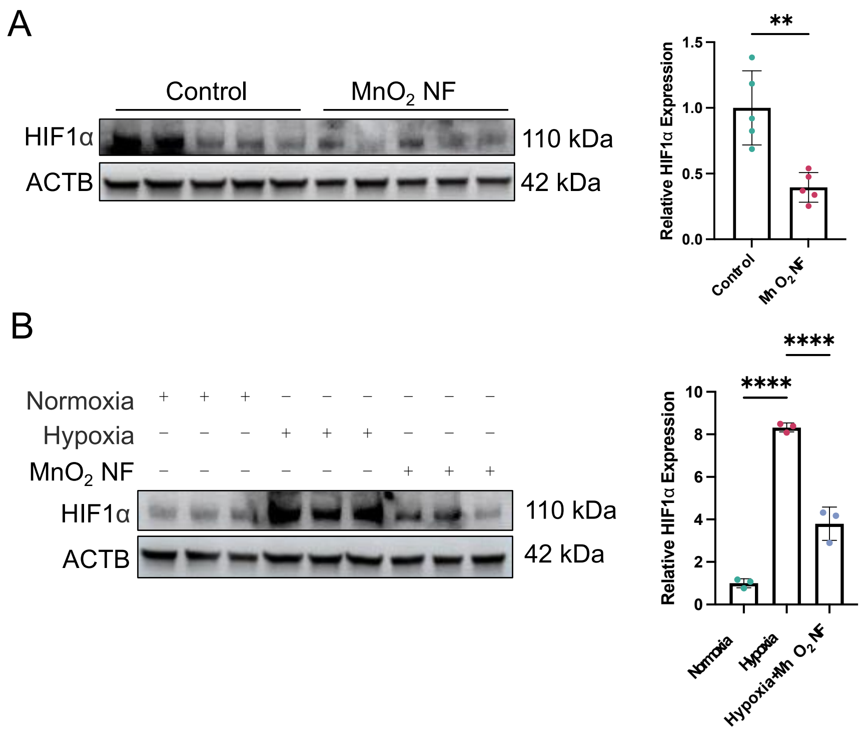

Figure S3. MnO_2 NFs mediate mitigation of the hypoxic tumor microenvironment. (**A**)Western blot analysis of HIF-1α expression in the tumor tissues (n = 3/group). (**B**)Western blot analysis of HIF-1α expression in Hepa1-6 cells (n = 3/group). Data were presented as mean ± SD. Ordinary one-way ANOVA and unpaired t-test were performed, $^{**}p < 0.01$, $^{****}p < 0.0001$.

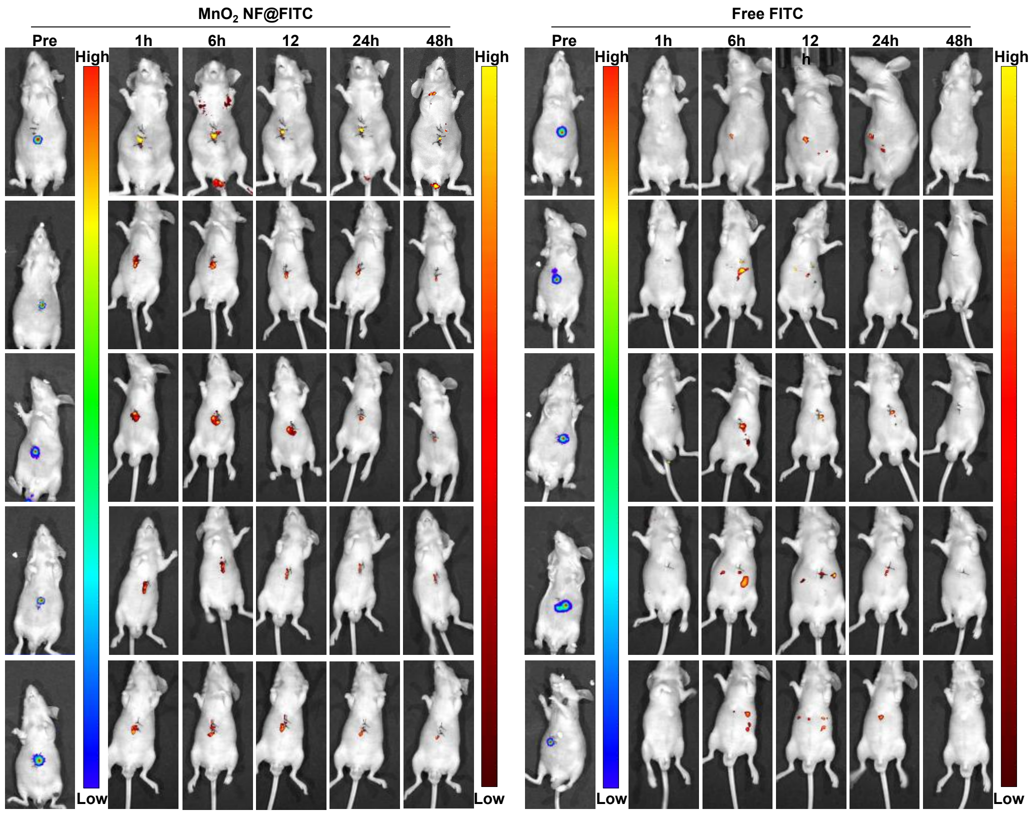

Figure S4. Time-dependent in vivo live imaging of FITC-labeled MnO_2 NFs in orthotopic Hepa1-6 tumors in mice.

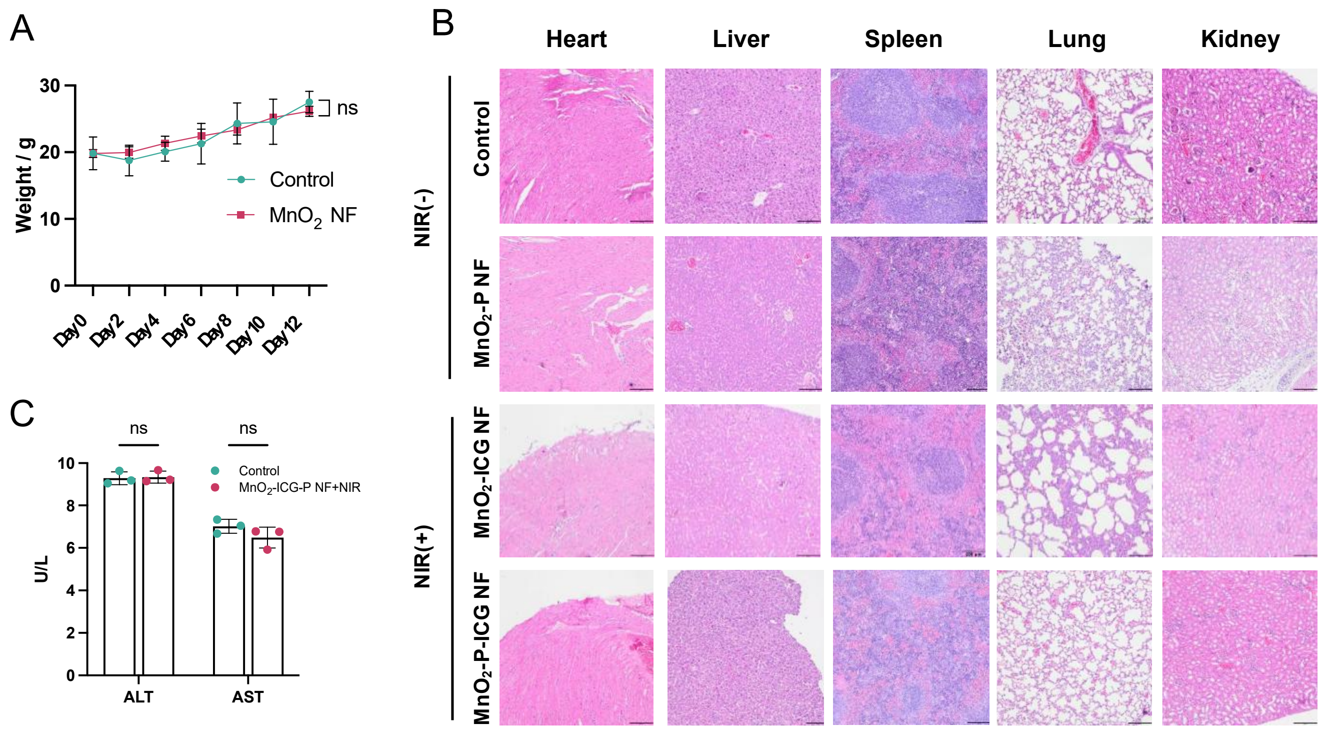

Figure S5. Biocompatibility of the composite drug delivery system carrier, MnO_2 NFs, in vivo.

(**A**) Changes in body weight of mice in the control group and MnO_2 NFs implantation group (*n* = 3/group). (**B**) Representative histopathological sections of major organs from mice in the control group and various groups (scale bar = 200 μm). (**C**) Changes in serum ALT and AST levels in mice from the control group and composite drug delivery system group (*n* = 3/group). Data were presented as mean ± SD. Unpaired t-test was performed, ns = not significant.

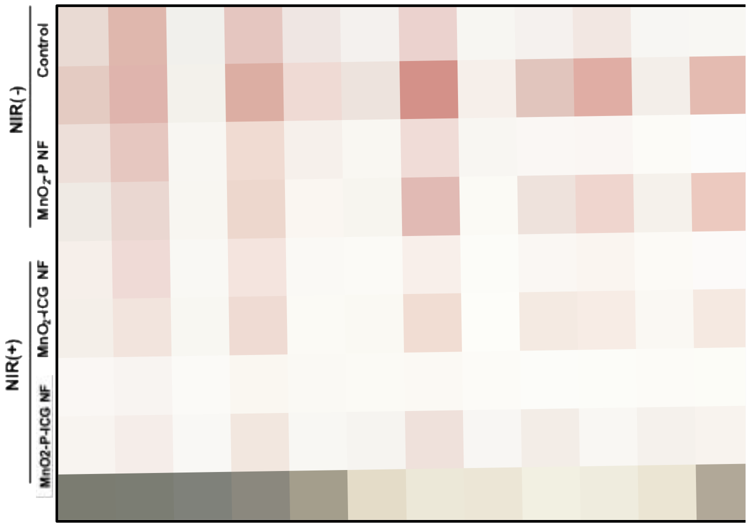

Figure S6. Gross images of orthotopic tumors in mice from each group post-treatment.

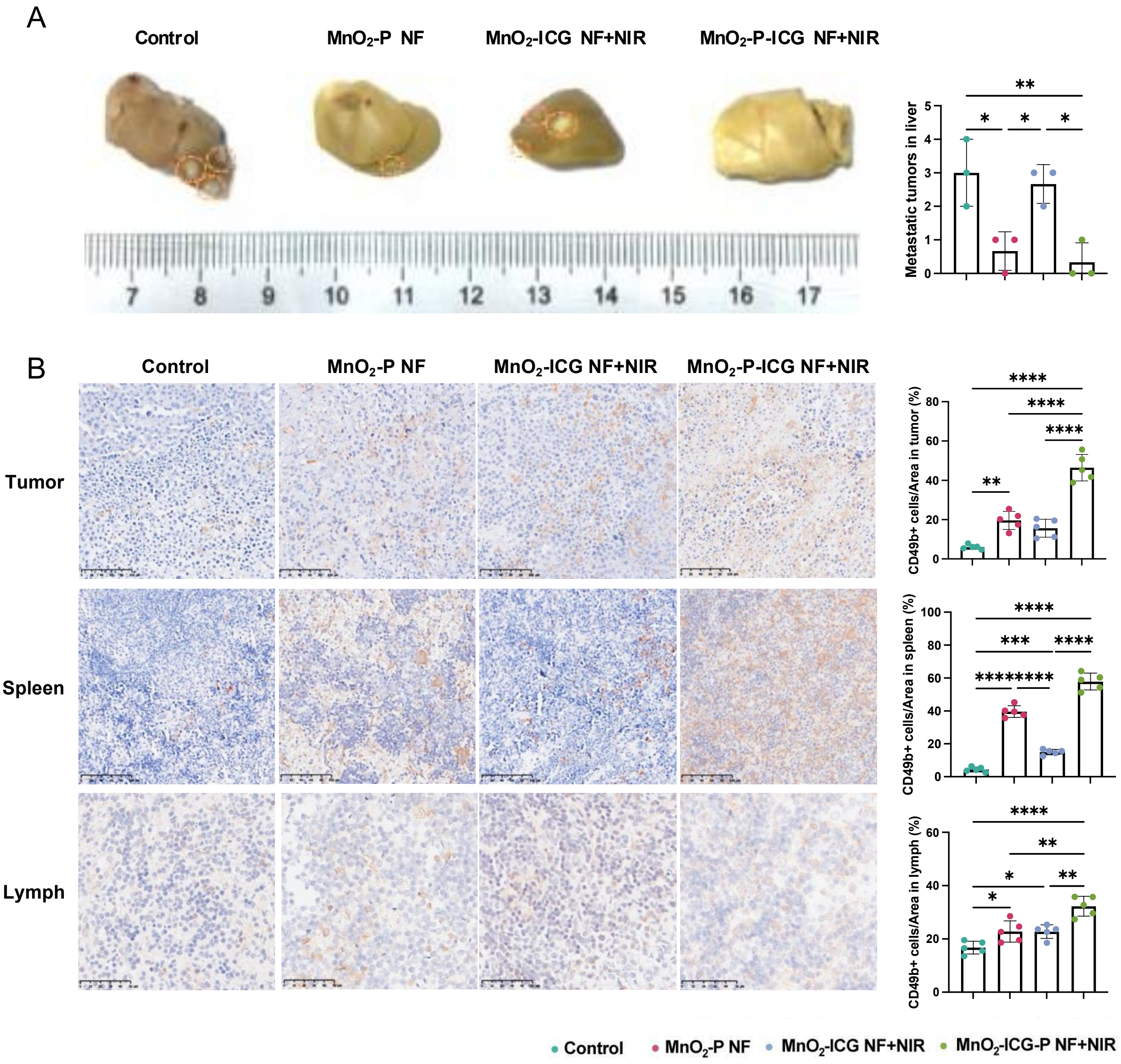

Figure S7. Gross and pathological images of tumors in mouse groups.

(**A**) Gross images of liver metastasis in mice from each group post-treatment (n = 3/group). (**B**) Representative IHC staining of CD49b in tumors, spleen, and abdominal lymph node tissues of mice from each group (scale bar = 100 μm, n = 5/group). Ordinary one-way ANOVA was performed, *$p < 0.05$, **$p < 0.01$, ***$p < 0.001$, ****$p < 0.0001$.

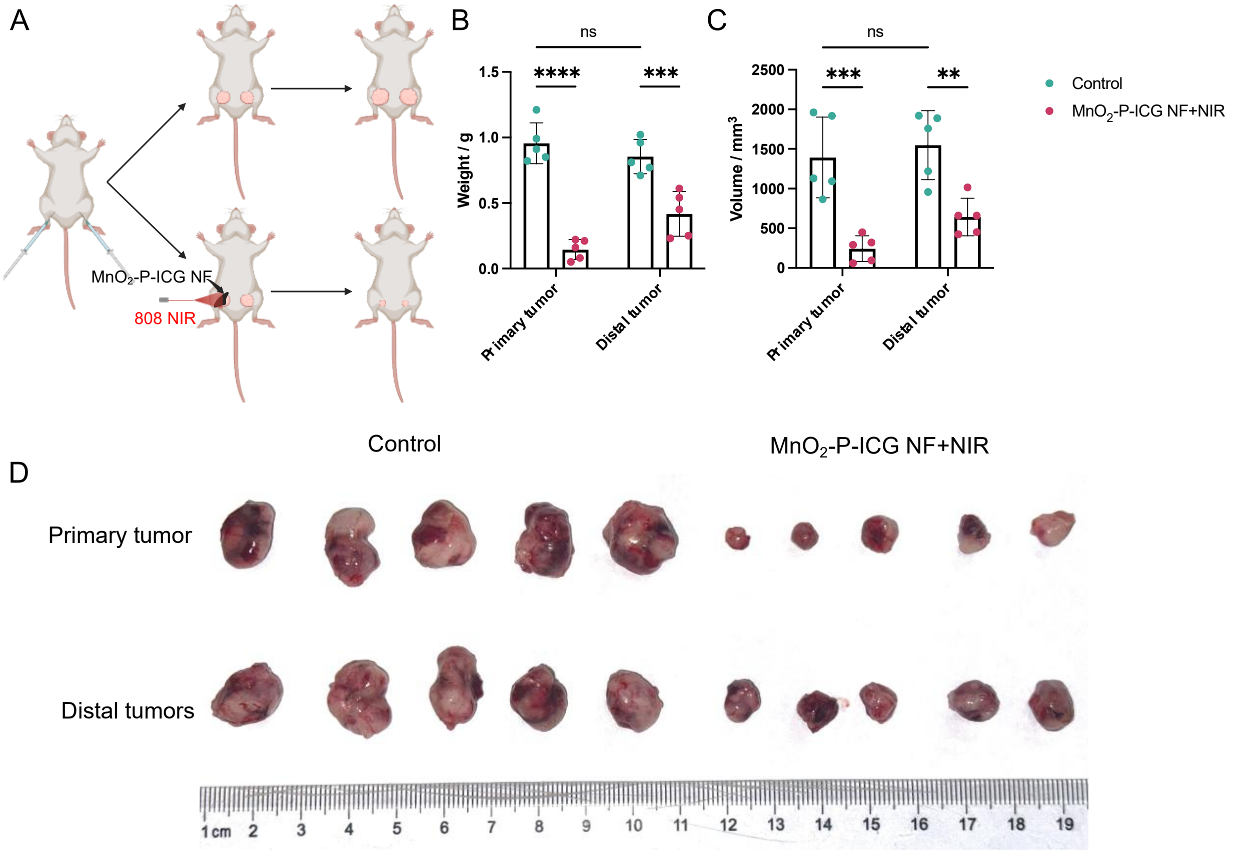

Figure S8. The composite patch triggers immune-mediated effects capable of suppressing distal tumor growth.

(A) Schematic diagram of the bilateral tumor model. (**B**) Tumor weight of mice post-treatment (*n* = 5/group). (**C**) Tumor volume of mice post-treatment (*n* = 5/group). (**D**) Gross images of primary and distal tumors in mice from each group post-treatment. Data were presented as mean ± SD. Two-way ANOVA was performed, $^{**}p < 0.01$, $^{***}p < 0.001$, $^{****}p < 0.0001$, ns = no significant.

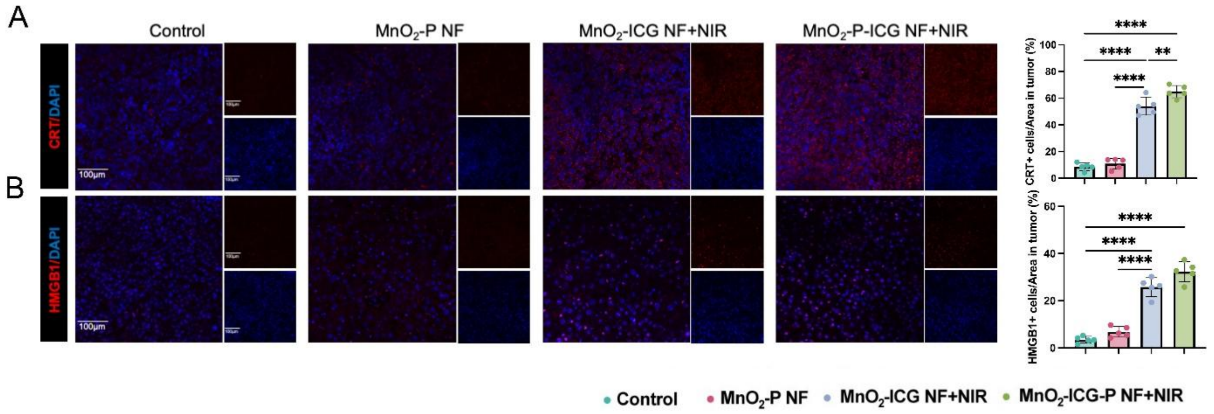

Figure S9. The effect of the composite drug delivery system MnO_2-P-ICG NFs on immunogenic cell death in vivo.

(**A**)Representative immunofluorescence images of CRT expression, the markers of ICD, in mouse tumor tissues (scale bar = 100 μm, *n* = 5/group). (**B**)Representative immunofluorescence images of HMGB1 expression, the markers of ICD, in mouse tumor tissues (scale bar = 100 μm, *n* = 5/group). Data were presented as mean ± SD. Ordinary one-way ANOVA was performed, $^{**}p < 0.01$, $^{****}p < 0.0001$.

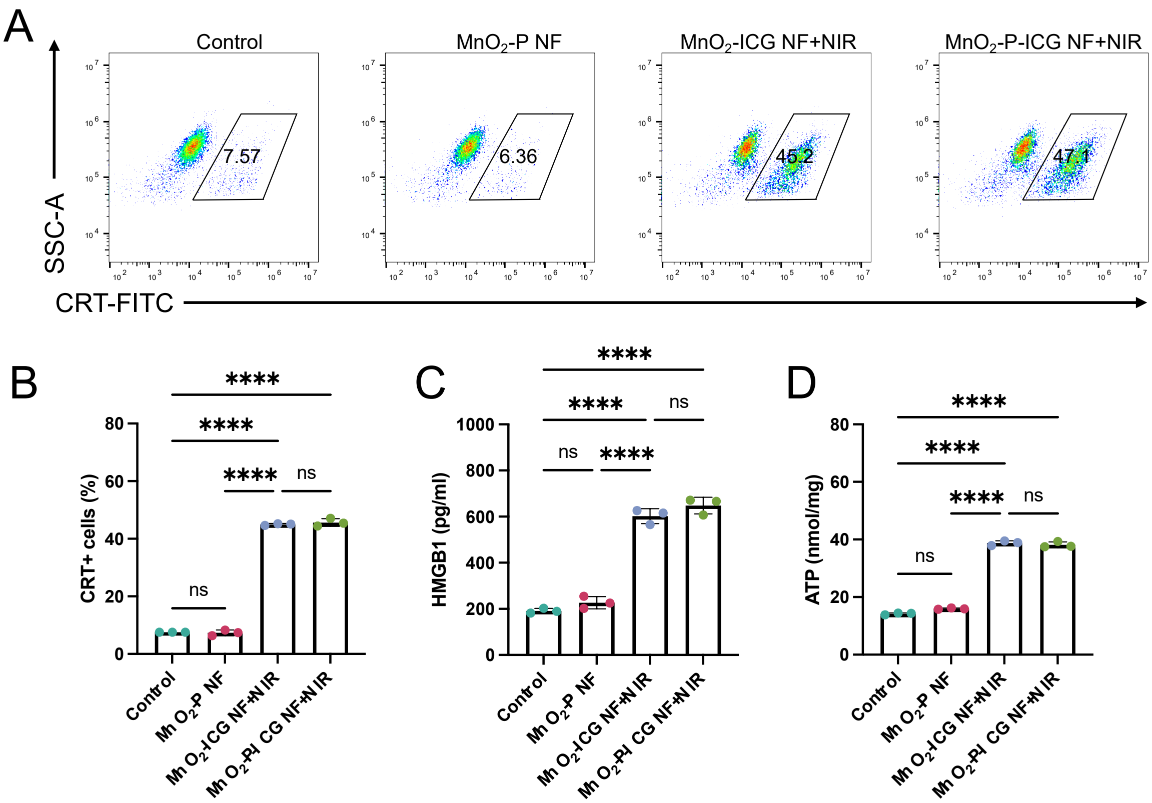

Figure S10. The effect of the composite drug delivery system MnO_2-P-ICG NFs on immunogenic cell death in vitro. (**A-B**) Representative flow cytometry analysis of CRT exposure, the markers of ICD, in Hepa1-6 cells (n = 3/group). (**C**) ELISA based quantification of HMGB1 levels in Hepa1-6 cells (n = 3/group). (**D**) Quantification of extracellular ATP release in Hepa1-6 cells (n = 3/group). Data were presented as mean ± SD. Ordinary one-way ANOVA was performed, ****$p < 0.0001$, ns = not significant.

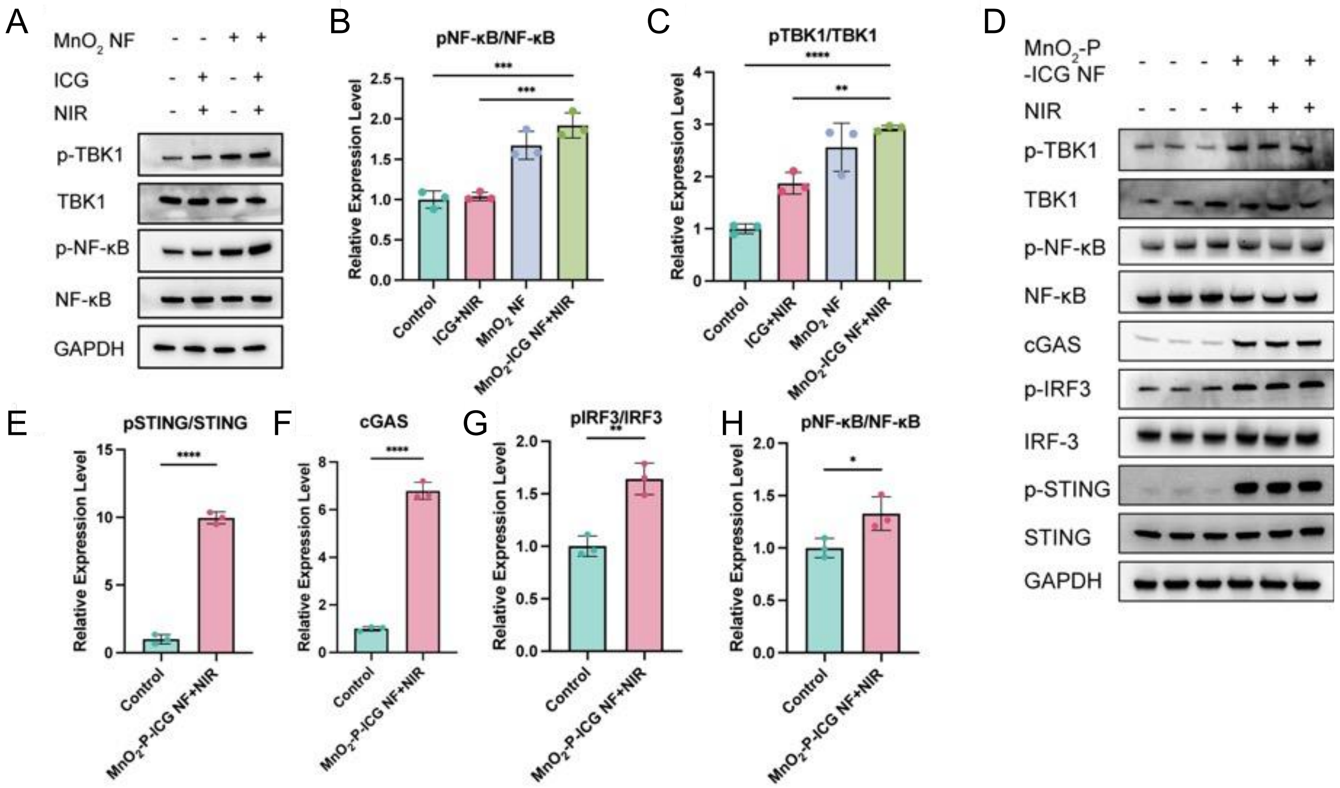

Figure S11. Results of the cGAS-STING pathway protein western blot experiment. (**A**) Western blot results of the cGAS-STING pathway proteins in the Hepa1-6 cell line. (**B**) Expression levels of pNF-κB/NF-κB in the Hepa1-6 cell line (n = 3/group). (**C**) Expression levels of pTBK1/TBK1 in the Hepa1-6 cell line (n = 3/group). (**D**) Western blot results of the cGAS-STING pathway proteins in the tumor tissues of the control group and the composite drug delivery system group. (**E**) Expression levels of pSTING/STING in the tumor tissues (n = 3/group). (**F**) Expression levels of cGAS in the tumor tissues (n = 3/group). (**G**) Expression levels of pIRF3/IRF3 in the tumor tissues (n = 3/group). (**H**) Expression levels of pNF-κB/NF-κB in the tumor tissues (n = 3/group). Data were presented as mean ± SD. Ordinary one-way ANOVA and unpaired t-test were performed, $^*p < 0.05$, $^{**}p < 0.01$, $^{***}p < 0.001$, $^{****}p < 0.0001$.

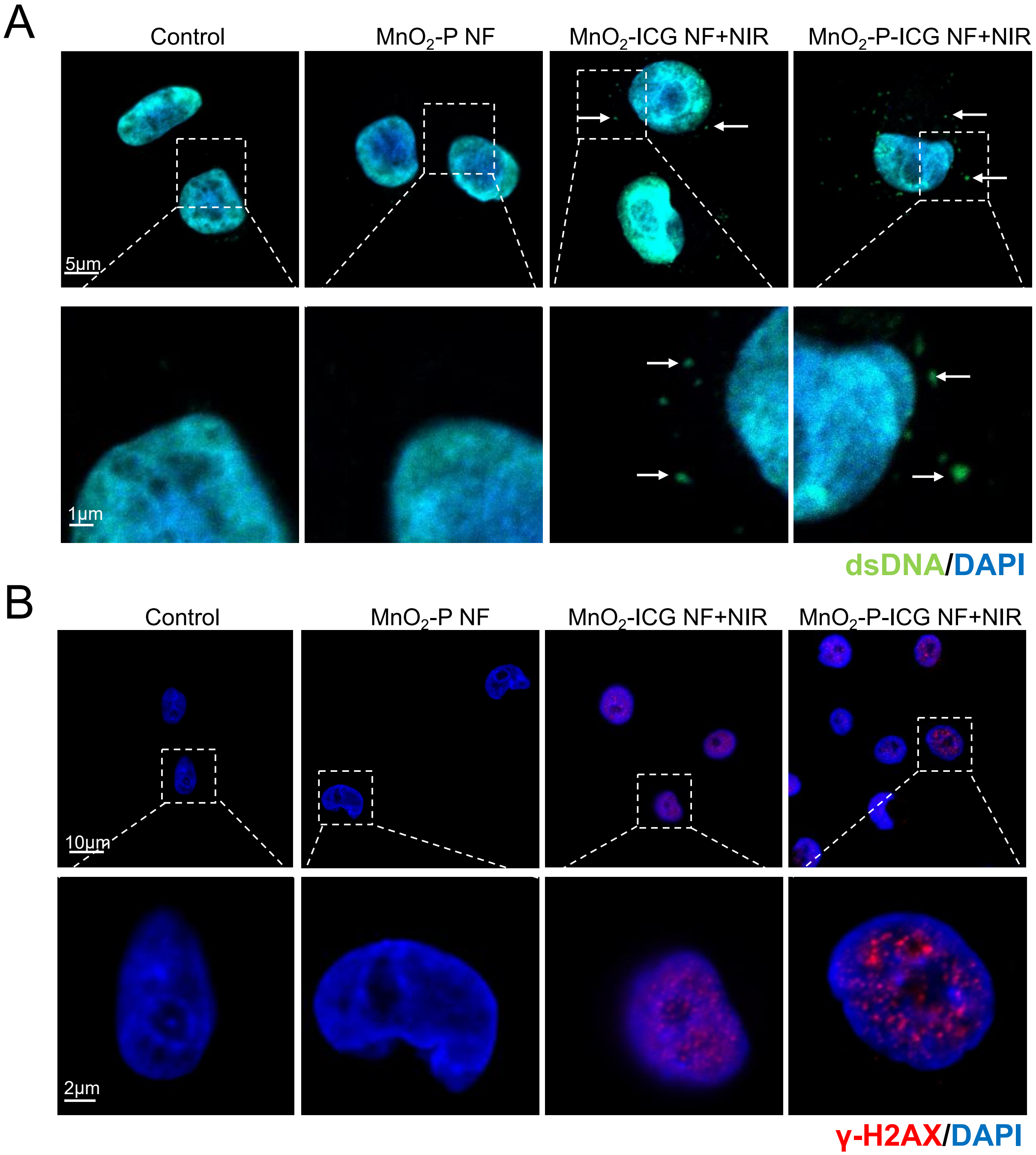

Figure S12. Immunofluorescence analysis showing patch-induced dsDNA accumulation and DNA damage in tumor cells. (**A**) Representative immunofluorescence images of dsDNA accumulation in Hepa1-6 cells (scale bar = 5 μm, 1 μm). (**B**) Representative immunofluorescence images of DNA damage in Hepa1-6 cells (scale bar = 10 μm, 2 μm).

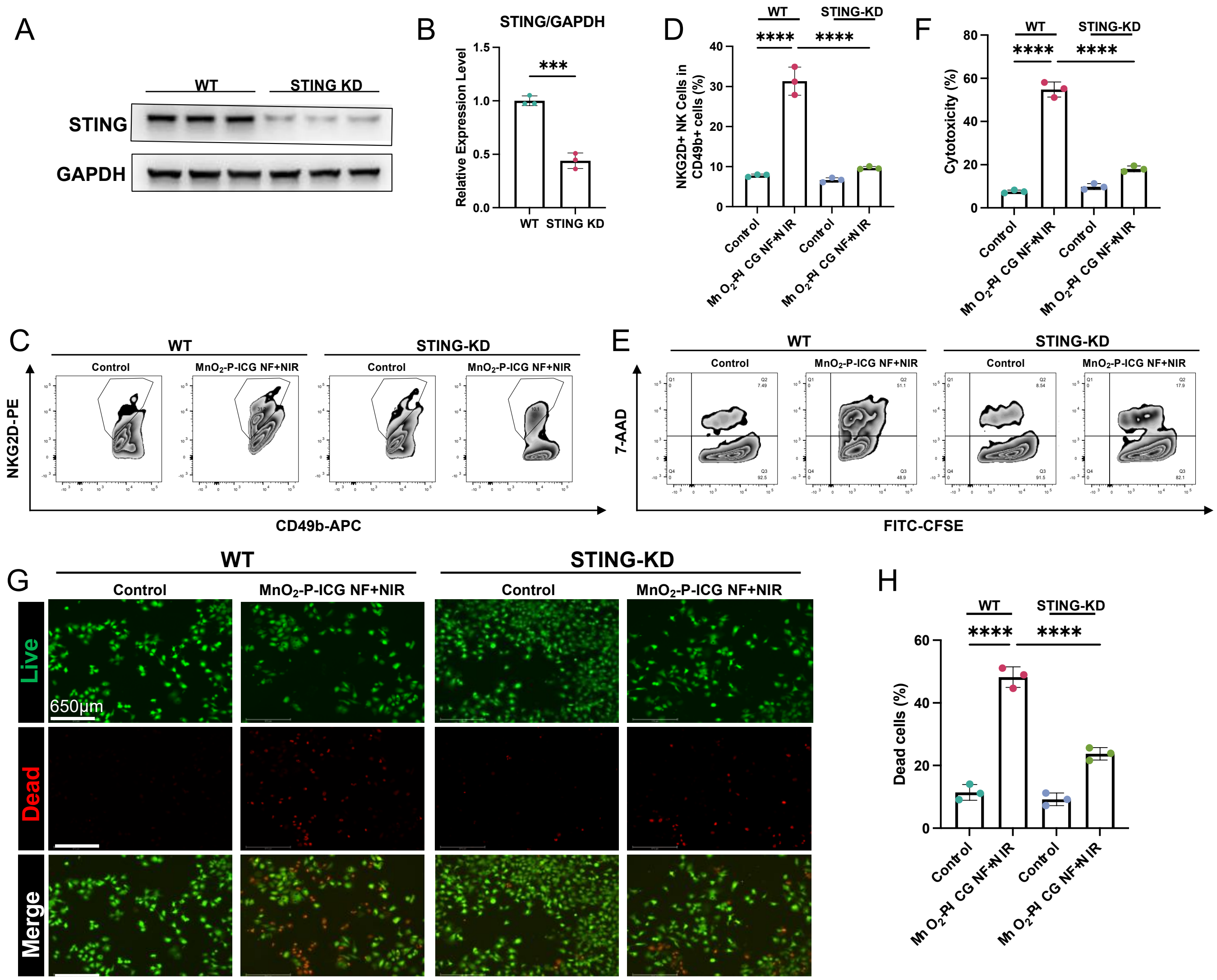

Figure S13. STING inhibitor impairs the therapeutic effect of the composite drug delivery system MnO2-P-ICG NFs in vitro. (**A, B**) Western blot results of the knockdown efficiency of STING in Hepa1-6 cells (*n* = 3/group). (**C, D**) Flow cytometry analysis of the activation effect of STING inhibitor on NK cells (*n* = 3/group). (**E, F**) Flow cytometry analysis of the effect of STING inhibitor on NK cell cytotoxicity (*n* = 3/group). (**G, H**) Live/Dead staining showing the proportion of NK cell cytotoxicity impaired by STING inhibitor (scale bar = 650 µm, *n* = 3/group). Data were presented as mean ± SD. Ordinary one-way ANOVA was performed, ***$p < 0.001$, ****$p < 0.0001$.

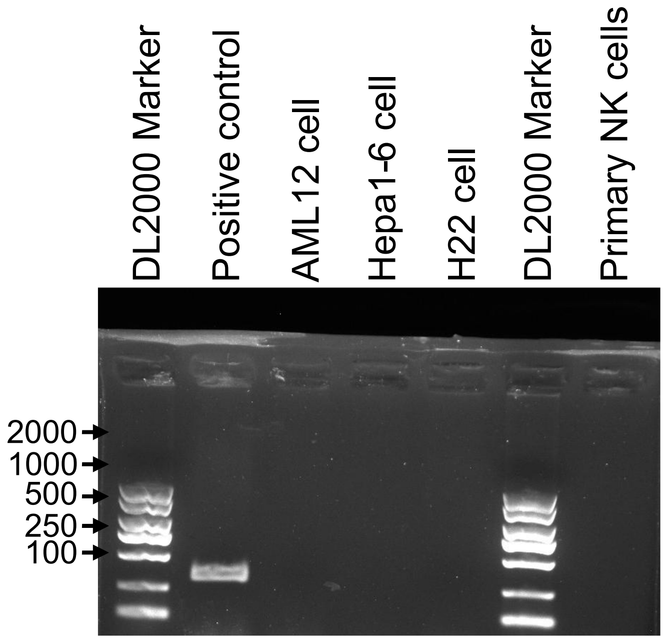

Figure S14. The most recent mycoplasma test results using PCR.

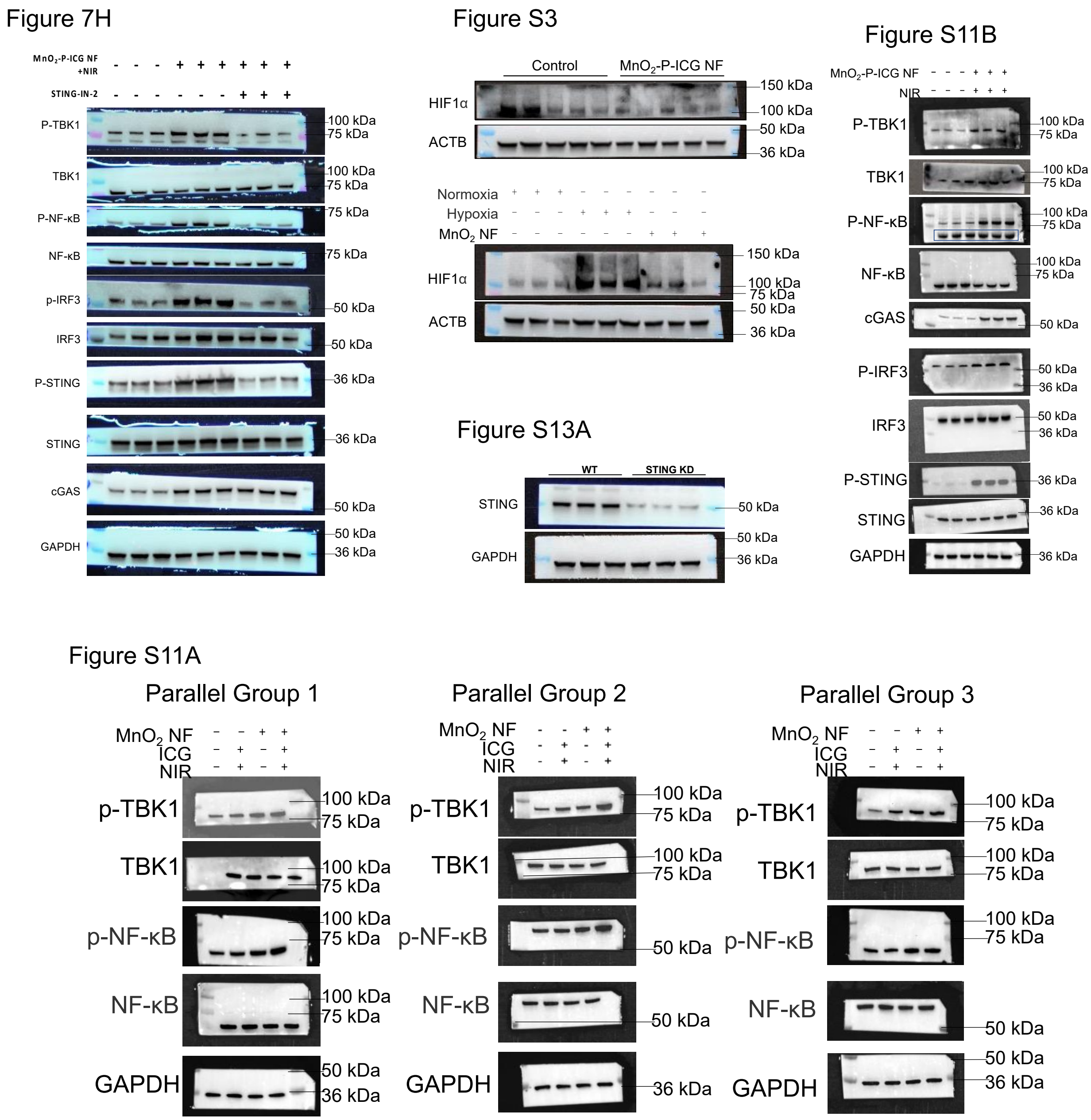

Figure S15. The raw data of western blot images.

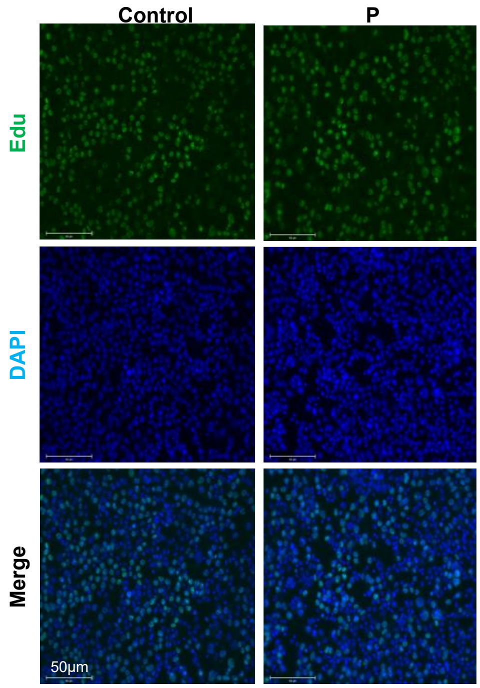

Figure S16. Original uncropped immunofluorescence images corresponding to Figure 1O.

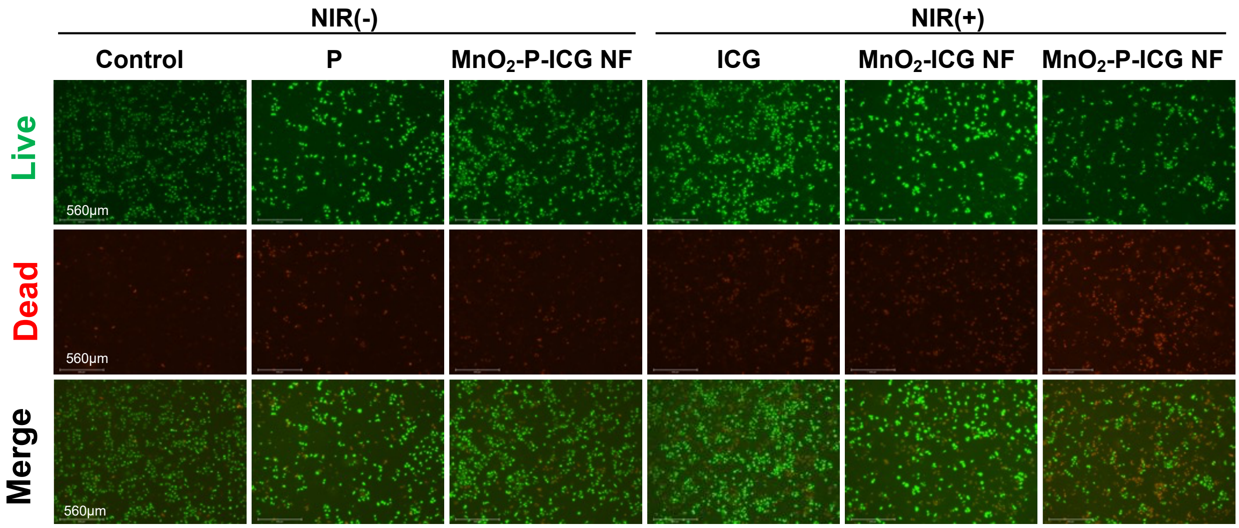

Figure S17. Original uncropped immunofluorescence images corresponding to Figure 3C.

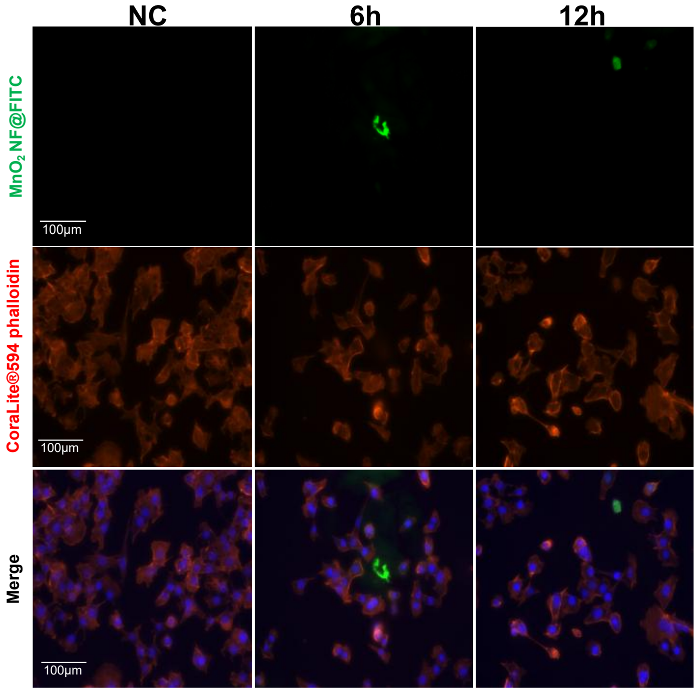

Figure S18. Original uncropped immunofluorescence images corresponding to Figure 4G.

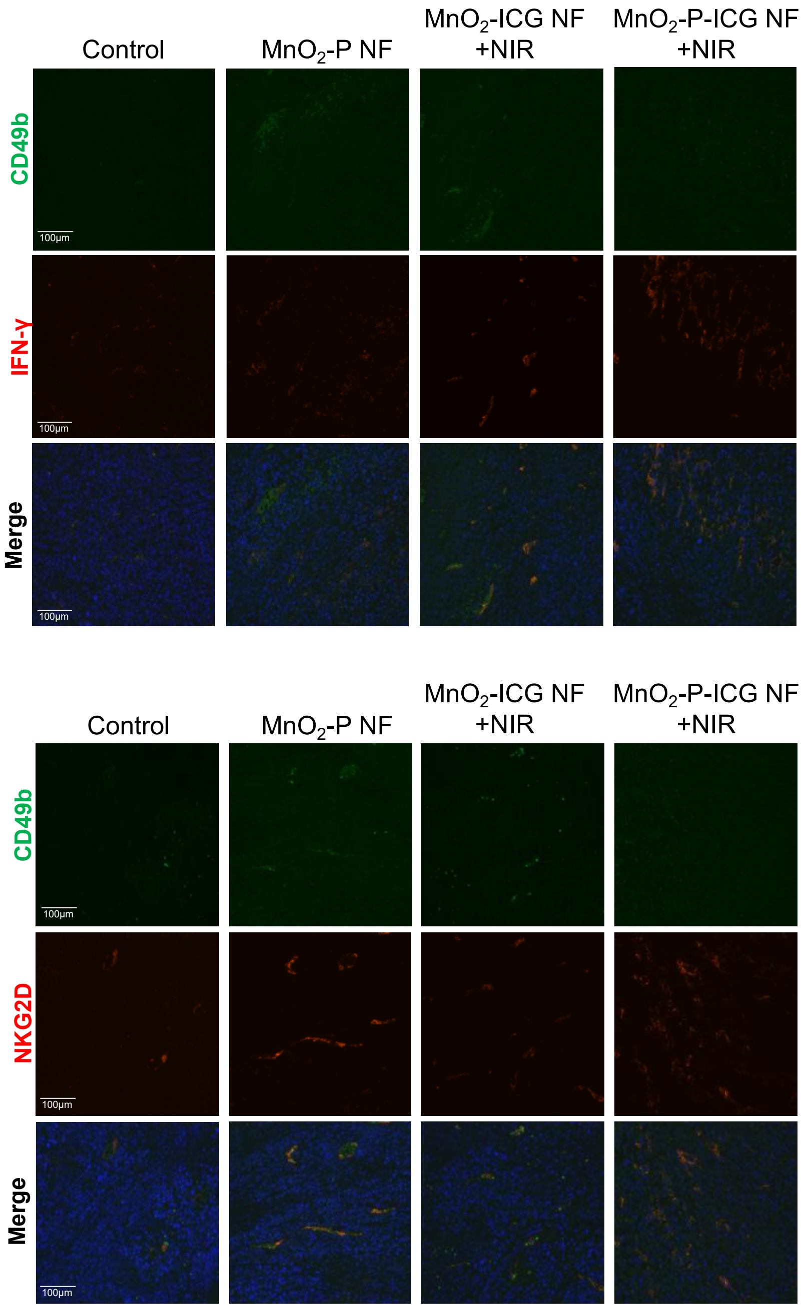

Figure S19. Original uncropped immunofluorescence images corresponding to Figure 6G.

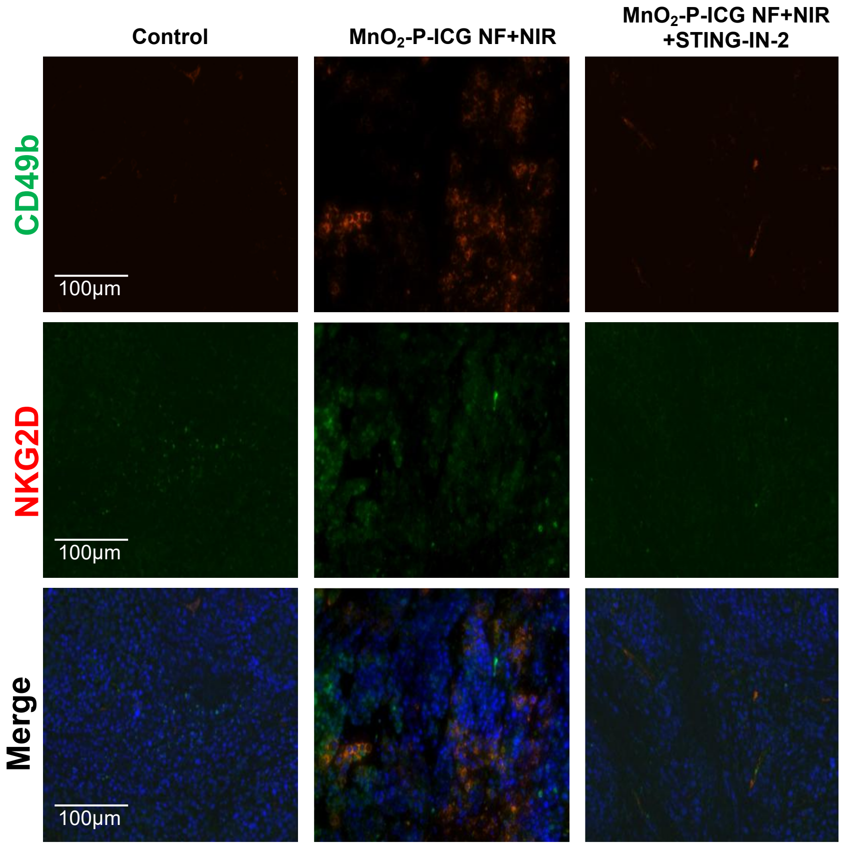

Figure S20. Original uncropped immunofluorescence images corresponding to Figure 7F.

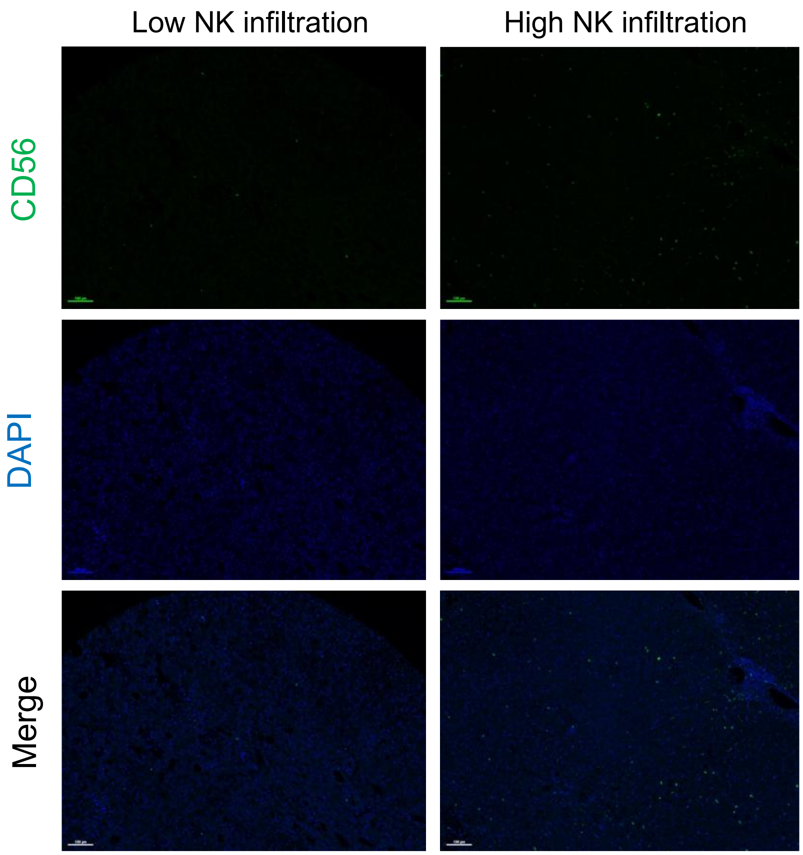

Figure S21. Original uncropped immunofluorescence images corresponding to Figure 8A.

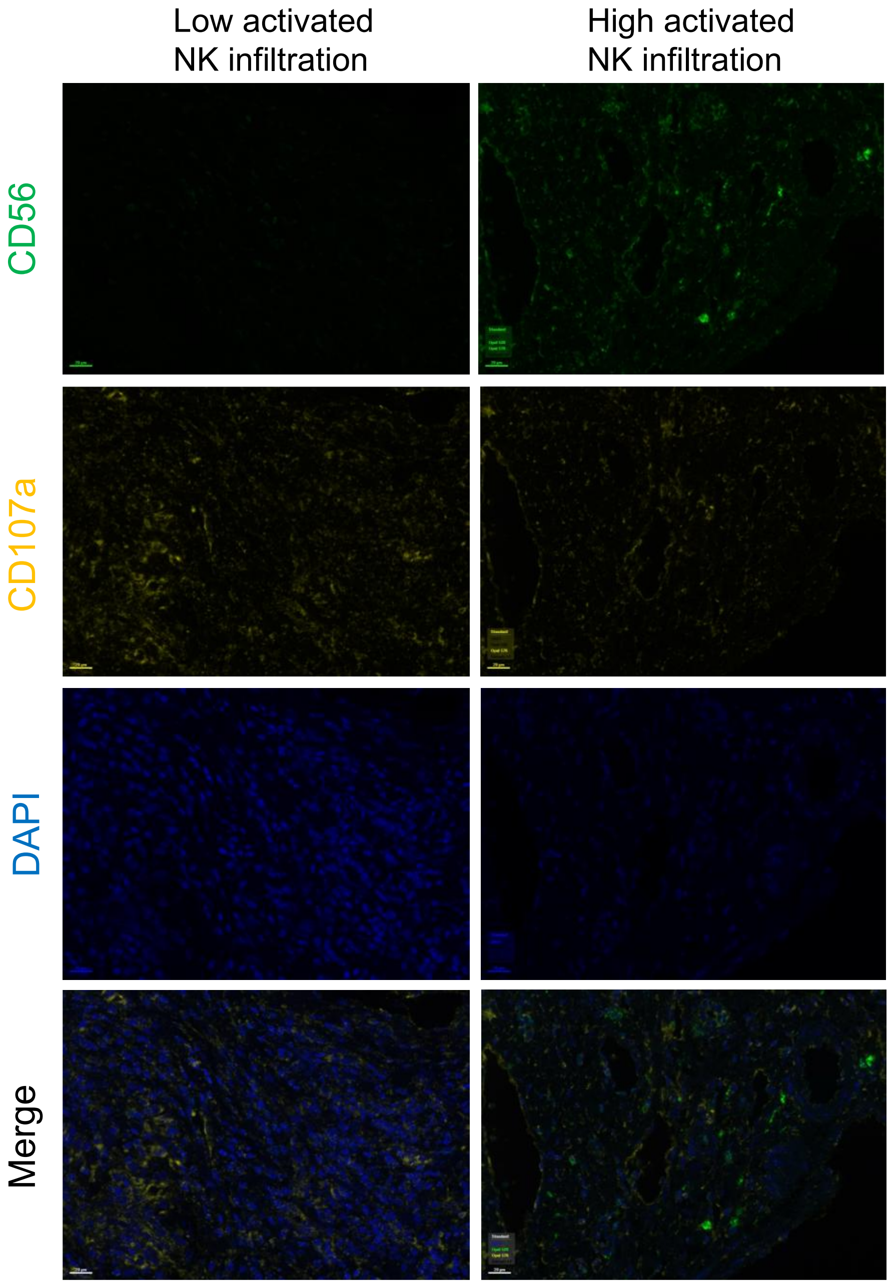

Figure S22. Original uncropped immunofluorescence images corresponding to Figure 8B.

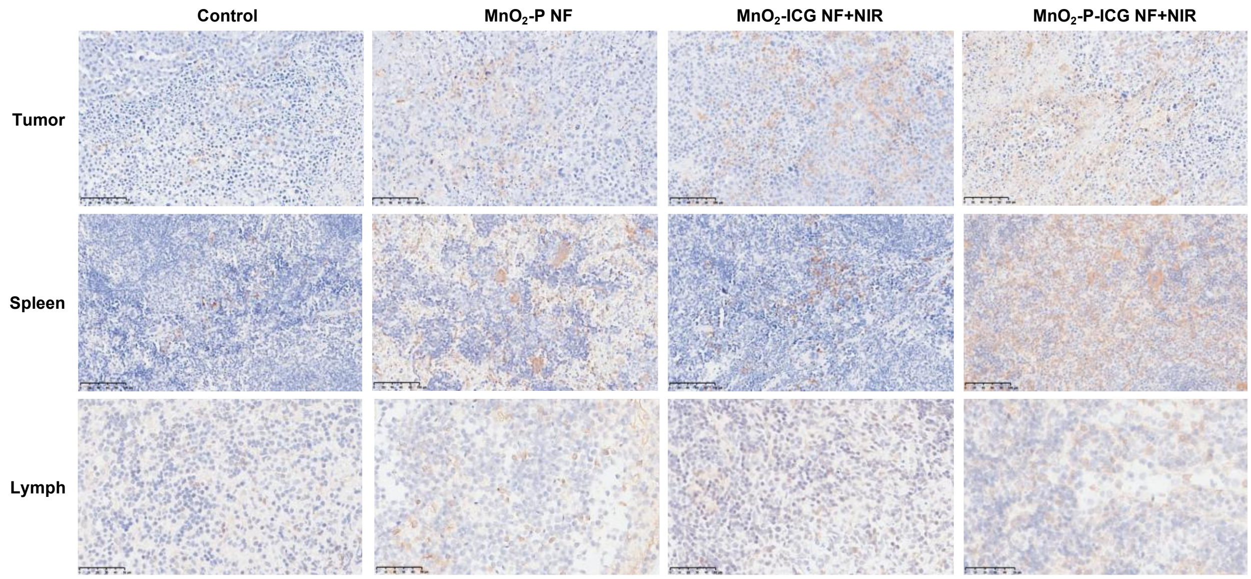

Figure S23. Original uncropped immunofluorescence images corresponding to Figure S7B.

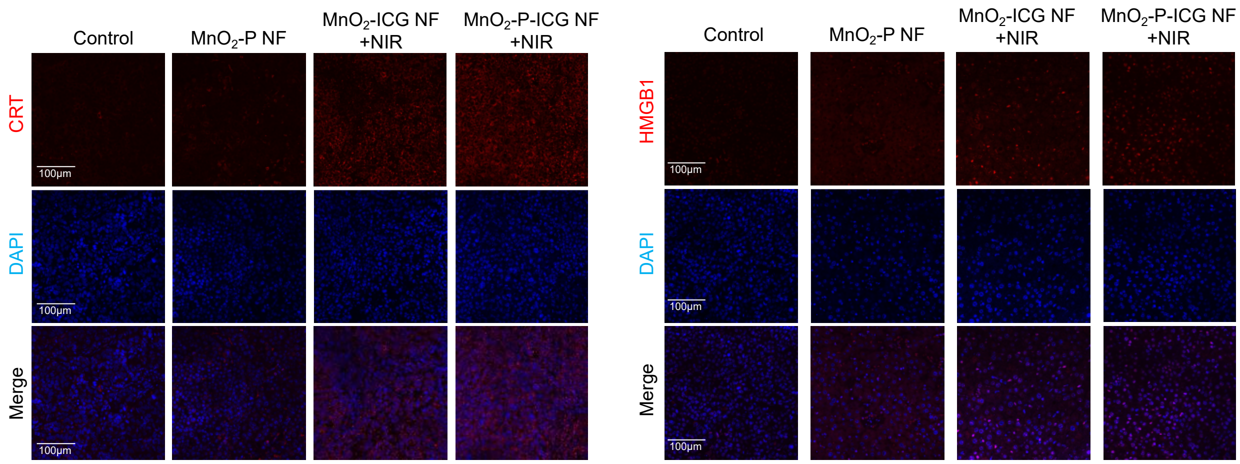

Figure S24. Original uncropped immunofluorescence images corresponding to Figure S9.

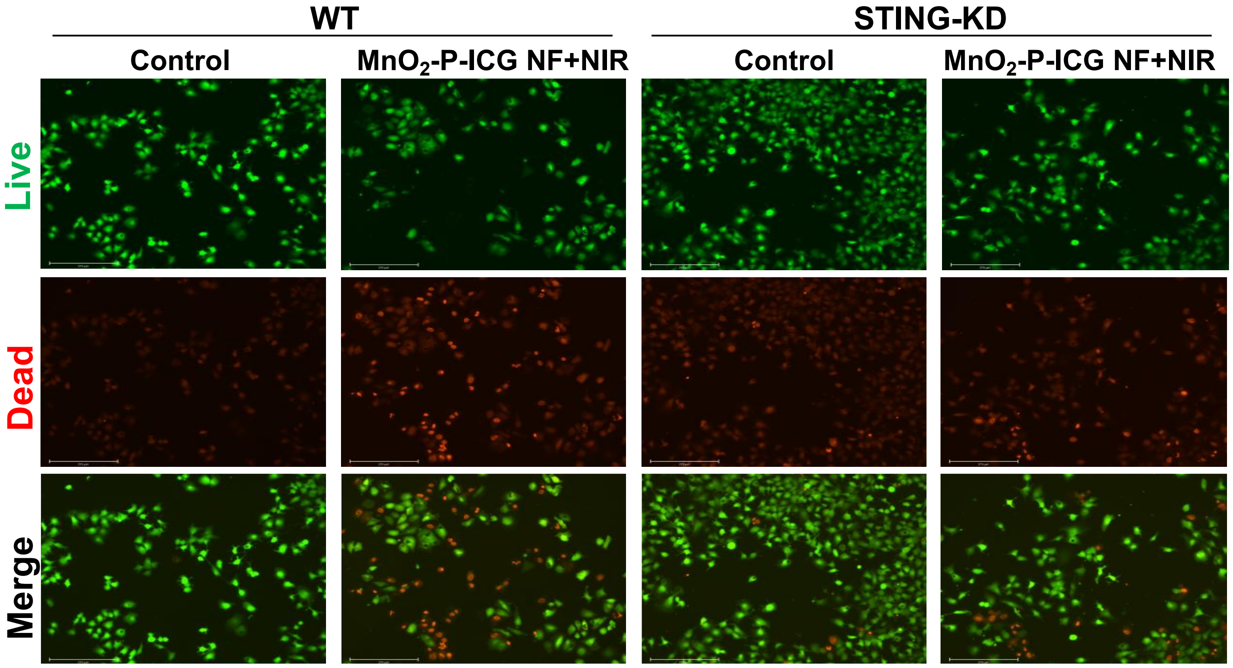

Figure S25. Original uncropped immunofluorescence images corresponding to Figure S13G.

Data S1. (separate file)
The RNA-seq of tumor samples.

Data S2. (separate file)
A gene set for immune traits of mice.

Table S1. Clinical characteristics of HCC patients enrolled in the cohort.

Variable	Total patients (n=24)	High NK infiltration Group (n=12)	Low NK infiltration Group (n=12)	High activated NK infiltration Group (n=12)	Low activated NK infiltration Group (n=12)
**Demographic Characteristics**					
Age (years), median (range)	52 (34-71)	60.5 (44–71)	52 (34–68)	55.5 (34–71)	54 (34–68)
Sex, n (%)					
Male	20 (83.3%)	8 (66.7%)	12 (100%)	8 (66.7%)	12 (100%)
Female	4 (16.7%)	4 (33.3%)	0 (0%)	4 (33.3%)	0 (0%)
**Pathological Characteristics**					
Edmondson grade, n (%)					
II	7 (25.0%)	4 (33.3%)	3 (25.0%)	5 (41.7%)	2 (16.7%)
III	18 (64.3%)	7 (58.3%)	7 (58.3%)	6 (50.0%)	7 (58.3%)
IV	3 (10.7%)	1 (8.3%)	2 (16.7%)	1 (8.3%)	7 (58.3%)
Microvascular invasion (MVI) grade, n (%)					
M0	11 (39.3%)	6 (50.0%)	3 (25.0%)	7 (58.3%)	2 (16.7%)
M1	10 (35.7%)	6 (50.0%)	4 (33.3%)	5 (41.7%)	5 (41.7%)
M2	7 (25.0%)	0 (0%)	5 (41.7%)	0 (0%)	5 (41.7%)

**Tumor Characteristics**					
Vascular invasion, n (%)	14 (58.3%)	6 (50.0%)	8 (66.7%)	4 (33.3%)	9 (75.0%)
(Microscopic) Vascular tumor thrombus, n (%)	14 (58.3%)	6 (50.0%)	8 (66.7%)	5 (41.7%)	9 (75.0%)
Satellite nodules, n (%)	3 (12.5%)	0 (0%)	3 (25.0%)	0 (0%)	3 (25.0%)
Multiple tumors, n (%)	5 (20.8%)	2 (16.7%)	3 (25.0%)	2 (16.7%)	3 (25.0%)

Table S2. The detailed information of antibodies

Names	Cat No.	Company
anti-cGAS(E5V3W)	Cat# 79978T	Cell Signaling Technology
anti-Phospho-STING (Ser366) (D7C3S)	Cat# 19781T	Cell Signaling Technology
anti-STING (D2P2F)	Cat# 13647T	Cell Signaling Technology
anti-GAPDH (D4C6R)	Cat# 97166T	Cell Signaling Technology
anti-NF-κB p65 (D14E12)	Cat# 8242T	Cell Signaling Technology
anti-Phospho-NF-κB p65 (Ser536) (93H1)	Cat# 3033T	Cell Signaling Technology
anti-IRF-3 (D83B9)	Cat# 4302S	Cell Signaling Technology
anti-Phospho-IRF-3 (Ser396) (4D4G)	Cat# 4947S	Cell Signaling Technology
anti-TBK1/NAK (D1B4)	Cat# 3504T	Cell Signaling Technology
anti-Phospho-TBK1/NAK (Ser172) (D52C2)	Cat# 5483T	Cell Signaling Technology
FITC anti-mouse CD45	Cat# 147709	Biolegend
Brilliant Violet 421™ anti-mouse CD3	Cat# 100228	Biolegend
APC anti-mouse CD49b	Cat# 108910	Biolegend
PE anti-mouse CD314 (NKG2D)	Cat# 130208	Biolegend
APC anti-mouse NK-1.1 Antibody	Cat# 156506	Biolegend
TruStain FcX™ PLUS (anti-mouse CD16/32)	Cat# 156604	Biolegend
anti-NKG2D	Cat# ab203353	Abcam
anti-Interferon gamma[EPR21704]	Cat# ab231036	Abcam
anti-Integrin alpha 2[EPR17338]	Cat# ab181548	Abcam
anti-HMGB1	Cat# ab18256	Abcam
anti-Calreticulin[EPR3924]	Cat# ab92516	Abcam
